# Metabolic bariatric surgery is associated with reduced adverse hepatic and extrahepatic outcomes, and lower all‐cause mortality, in patients with steatotic liver disease

**DOI:** 10.1111/dom.70173

**Published:** 2025-09-30

**Authors:** Weronika Stupalkowska, Alexander Henney, Eric G. Sheu, Uazman Alam, Daniel J. Cuthbertson

**Affiliations:** ^1^ Department of Cardiovascular and Metabolic Medicine Institute of Life Course and Medical Sciences, University of Liverpool Liverpool UK; ^2^ Laboratory for Surgical and Metabolic Research, Department of Surgery Brigham and Women's Hospital Boston Massachusetts USA; ^3^ Metabolism & Nutrition Research Group Liverpool University Hospitals NHS Foundation Trust Liverpool UK; ^4^ Division of General and GI Surgery Brigham and Women's Hospital Boston Massachusetts USA; ^5^ Liverpool Centre for Cardiovascular Sciences University of Liverpool and Liverpool University Hospitals NHS Foundation Trust Liverpool UK; ^6^ Centre for Biomechanics and Rehabilitation Technologies Staffordshire University Stoke‐on‐Trent UK

## Abstract

**Aim:**

Metabolic bariatric surgery (MBS) improves histological endpoints in steatotic liver disease (SLD), but data on longer‐term clinical outcomes in this population are scarce. Here, we assessed the impact of MBS on hepatic and extrahepatic morbidity and mortality in individuals with SLD.

**Methods:**

Patients with SLD, with/without a history of MBS (MBS/no‐MBS cohorts, respectively) between 01/01/2004 and 31/10/2019, were identified using the TriNetX platform. Cohorts were balanced with propensity score matching (PSM). Maximum follow‐up was set to 5 years. *The primary outcome* was a composite of major adverse liver outcomes (MALO): cirrhosis, decompensated cirrhosis, hepatocellular carcinoma, and liver transplant. *Secondary outcomes* included major cardiovascular (MACE) and kidney (MAKE) adverse events, obesity‐associated cancers, and all‐cause mortality (ACM). We performed sub‐group analyses according to sex, MBS type, and risk factors (BMI ≥50 kg/m^2^ and type 2 diabetes (T2D)).

**Results:**

We identified 15,262 and 540,031 patients (for the MBS and no‐MBS cohorts, respectively); 14,970 patients/cohort after PSM (mean age: 46.7 vs. 47.4; female: 74.3% vs. 75.7%; mean follow‐up, 4.1 years). MBS was associated with reduced HR of MALO (0.84, 95% CI 0.75–0.95), MACE (0.52, CI 0.47–0.57), MAKE (0.54, CI 0.41–0.72), obesity‐related cancers (0.58, CI 0.50–0.67), and ACM (0.49, 0.43–0.56). In subgroup analyses, MBS was associated with reduced HR of MALO, MACE, MAKE, obesity‐related cancers, and ACM in females, patients with T2D, BMI > 50 kg/m^2^ and irrespective of surgery type.

**Conclusion:**

In patients with SLD, MBS is associated with significant reductions in the rates of adverse hepatic and extrahepatic outcomes and all‐cause mortality over 4 years' follow‐up.

## 1 INTRODUCTION

Despite potent and evolving pharmacotherapy, metabolic bariatric surgery (MBS) remains the most effective treatment for patients living with obesity,^1^ with an estimated 18.8%–25.5% total weight loss 5 years postoperatively.^2^ In addition to durable weight reduction, MBS is strongly associated with glycemic improvement, lower haemoglobin A1c (HbA1c), prescription of fewer glucose‐lowering medications, and a greater remission rate of type 2 diabetes (T2D), as compared to medical intervention.^3^ Moreover, via improvements in various traits of the metabolic syndrome (hypertension, dyslipidemia, etc.), MBS leads to a reduction in cardiovascular risk, a lower incidence of obesity‐associated cancers, and reduced all‐cause mortality (ACM).^4^

Obesity, T2D, and cardiometabolic risk factors strongly impact the natural progression of steatotic liver disease (SLD).^5^ Patients with SLD have a higher ACM, increased risks of major adverse liver outcomes, cardiovascular complications, chronic kidney disease, and specific extrahepatic cancers.^6^, ^7^, ^8^ Alarmingly, the prevalence of SLD continues to rise exponentially.^6^ This creates an urgent need for effective lifestyle interventions, pharmacological, and surgical therapies to prevent and reverse this condition and reduce the burden of its complications.

MBS not only ameliorates metabolic diseases co‐existing with SLD, but also achieves histological improvements within the liver, such as resolution of steatohepatitis and reduction in hepatic fibrosis.^9^, ^10^, ^11^ Improvement in these liver histological endpoints is associated with lower ACM.^12^ A large retrospective study on 1,158 patients treated with MBS, with biopsy‐proven steatohepatitis and fibrosis, demonstrated significantly reduced risk of adverse hepatic and cardiovascular outcomes.^13^ Nevertheless, data on clinical outcomes in SLD patients after MBS are scarce and come mostly from observational analyses, likely due to the fact that these studies require a large number of patients with extensive follow‐up.^14^ Here, we examined the impact of MBS on major hepatic and extrahepatic outcomes and ACM specifically in patients with non‐cirrhotic SLD using real‐world healthcare data.

## 2 METHODS

### 2.1 Study design

**FIGURE 1:**
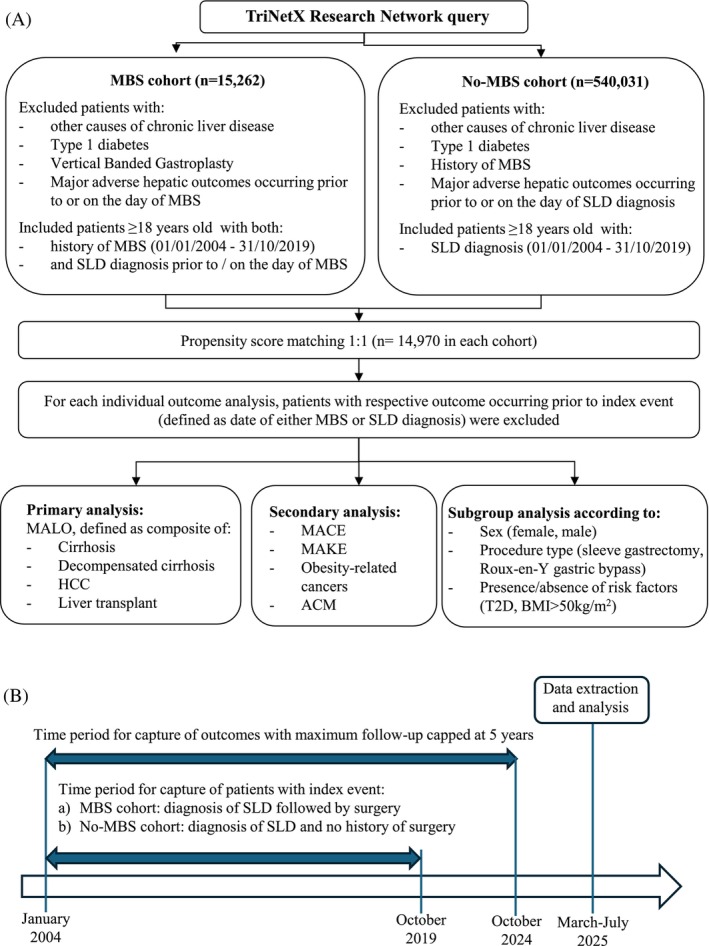
(A) Flow diagram to illustrate cohort construction in the study. (B) Study timeline. ACM, all‐cause mortality; BMI, body mass index; HCC, hepatocellular carcinoma; MACE, major adverse cardiovascular outcomes; MAKE, major adverse kidney events; MALO, major adverse liver outcomes; MBS, metabolic bariatric surgery; SLD, steatotic liver disease; T2D, type 2 diabetes.

This retrospective cohort study was conducted on the Research Network within the TriNetX platform.^15^ Data used in this study was extracted in March and July 2025. Figure 1A illustrates the process of cohorts' construction.

### 2.2 Study population

We identified all patients ≥18 years old with a history of SLD, including those with metabolic dysfunction‐associated steatotic liver disease (MASLD) and with/without mild‐to‐moderate alcohol consumption (MetALD) (Table S1). Individuals with alcohol‐associated liver disease (ALD) and other causes of chronic liver disease have been excluded (Table S2).

Patients were categorized into treatment (MBS) and reference (no‐MBS) group dependent upon their MBS status (Tables S3 and S4).

The inclusion and exclusion of patients was determined based on codes from the International Classification of Diseases, 10th Edition, Clinical Modification (ICD‐10‐CM), ICD Procedure Coding System (ICD‐10‐PCS), Current Procedural Terminology (CPT), and Systematized Nomenclature of Medicine Clinical Terms (SNOMED).

### 2.3 Index event

The index event was defined as the date of MBS (MBS cohort) or the date of SLD diagnosis (no‐MBS cohort). Additionally, for the MBS cohort, the diagnosis of SLD must have occurred prior to or on the day of MBS. The index event must have occurred between 01/01/2004 and 31/10/2019.

### 2.4 Follow‐up

Follow‐up started on the first day after the index event. Patients were followed up until the first coding of the respective outcome. The end of follow‐up was set to 5 years from the date of the index event. Patients without the outcome were censored either at the end of follow‐up or after the patient's last known record, whichever occurred earlier. The mean follow‐up was calculated in situ within the TrinNetX. Figure 1B illustrates the timeline of the study.

### 2.5 Propensity score matching

Cohorts were propensity score matched (PSM), in a 1:1 ratio for several major risk factors associated with adverse hepatic, cardiovascular, renal events, and cancer.^16^, ^17^, ^18^ Additionally, we included biochemical measurements and medications related to metabolic health to ensure their balanced distribution across study cohorts (Table S5).

### 2.6 Outcomes

The *primary outcome* was a composite of *major adverse liver outcomes* (MALO)^19^: cirrhosis, decompensated cirrhosis, hepatocellular carcinoma (HCC), and liver transplant (Table S6). The *secondary outcomes* included *major adverse cardiovascular events* (MACE; a composite of acute myocardial infarction, stroke, and heart failure, Table S7), *major adverse kidney events* (MAKE; a composite of chronic kidney disease stage 5, end‐stage renal disease, and progression to dialysis, Table S8), *obesity‐associated cancers* (a composite of 13 obesity‐related cancers,^20^ Table S9), and all‐cause mortality (ACM). Additionally, for the MBS cohort, we examined rates of 30‐day postoperative complications (Table S10).

### 2.7 Handling of competing risks

To address the possibility of competing risks within the composite outcomes (e.g., cirrhosis and HCC), we performed separate time‐to‐event analyses for each component individually. Furthermore, to address the possibility of competing risks between the main outcomes of interest (e.g., ACM and MALO), we report cumulative incidence ((number of patients with an event of interest during the follow‐up period/total number of patients in the cohort) × 100%) for each outcome of interest.^21^

### 2.8 Subgroup analysis

For each outcome, we performed subgroup analyses according to sex, procedure type (sleeve gastrectomy (SG) and Roux‐en‐Y gastric bypass (RYGB)), and presence/absence of additional metabolic risk factors (T2D and BMI ≥50 kg/m^2^).

### 2.9 Statistical Analysis

Statistical analysis was performed in situ within the TriNetX platform. For each outcome, a log‐rank test was performed, and a hazard ratio (HR) was generated.

Further details on methods are given in the Supporting Information Appendix.

## 3 RESULTS

### 3.1 Study cohorts

TriNetX Research Network query identified a total of 15,262 patients with SLD and a history of MBS between 2004 and 2019 (MBS cohort) and 540,031 patients with a diagnosis of SLD occurring in the same time window and without a history of MBS (no‐MBS cohort) (Table 1). After PSM, the cohorts included 14 970 patients each. The cohorts were reduced further during the subsequent analyses because of the exclusion of patients who had the respective outcome of interest prior to MBS/SLD diagnosis (Figure 1). The final numbers of patients in each cohort are given in Table 2.

**TABLE 1:** Baseline characteristics of patients with SLD and with or without history of metabolic bariatric surgery.

Characteristic	Before PSM			After PSM		
	MBS *n* = 15 262	No‐MBS *n* = 540 031	SMD	MBS *n* = 14 970	No‐MBS *n* = 14 970	SMD
Age, mean (SD), years	47 [12]	52 [15]	0.411	47 [12]	47[14]	0.053
Female sex, No. (%)	11 362 (74.6)	276 775 (51.5)	0.492	11 129 (74.3)	11 328 (75.7)	0.031
Race & ethnicity, No. (%)						
Asian	121 (0.8)	30 159 (5.6)	0.276	121 (0.8)	102 (0.7)	0.015
Black or African American	1853 (12.2)	70 724 (13.2)	0.152	1808 (12.1)	1974 (13.2)	0.033
White	11 012 (72.3)	368 848 (68.7)	0.080	10 832 (72.4)	10 797 (72.1)	0.005
Hispanic or Latino	2051 (13.5)	70 724 (13.2)	0.009	2013 (13.4)	1823 (12.2)	0.038
Anthropometric and biochemical measurements, mean (SD)						
BMI, kg/m^2^	42.7 [8.9]	33.4 [7.6]	1.132	42.5 [8.8]	42.0 [8.9]	0.053
HbA_1C_, *%*	6.2 [1.3]	6.6 [1.8]	0.247	6.2 [1.3]	6.3 [1.6]	0.078
ALT, U/L	39 [45]	55 [81]	0.247	39 [45]	42 [73]	0.055
AST, U/L	32 [43]	44 [95]	0.162	33 [44]	35 [54]	0.052
Triglycerides, mg/dL	157 [103]	186 [219]	0.167	157 [104]	162 [141]	0.034
HDL, mg/dL	45 [14]	45 [17]	0.037	45 [14]	44 [16]	0.073
LDL, mg/dL	105 [36]	108 [39]	0.077	105 [36]	104 [36]	0.030
Comorbidities, No. (%)						
T2D	5090 (33.4)	104 568 (19.5)	0.320	4959 (33.1)	5208 (34.8)	0.035
Hypertension	9046 (59.4)	216 906 (40.4)	0.387	8832 (59.0)	9187 (61.4)	0.048
IHD	1195 (7.8)	46 145 (8.6)	0.027	1182 (7.9)	1265 (8.5)	0.020
Cerebrovascular disease	311 (2.0)	16 221 (3.0)	0.062	309 (2.1)	334 (2.2)	0.012
Dyslipidemia	7126 (46.8)	190 303 (35.4)	0.232	6966 (46.5)	7101 (47.4)	0.018
Smoking	1075 (7.1)	46 309 (8.6)	0.058	1066 (7.1)	1163 (7.8)	0.025
CKD	557 (3.7)	20 168 (3.8)	0.005	553 (3.7)	597 (4.0)	0.015

Medication, No. (%)						
Insulin	3038 (19.9)	36 666 (6.8)	0.393	2904 (19.4)	2964 (19.8)	0.010
Oral Hypoglycemic^a^	2684 (17.6)	61 496 (11.4)	0.176	2632 (17.6)	2775 (18.5)	0.025
Other Hypoglycemic^b^	466 (3.1)	5494 (1.0)	0.144	451 (3.0)	442 (3.0)	0.004
ACE inhibitors	2307 (15.1)	62 079 (11.6)	0.106	2261 (15.1)	2380 (15.9)	0.022
Angiotensin II inhibitors	1432 (9.4)	40 498 (7.5)	0.067	1408 (9.4)	1488 (9.9)	0.018
Beta‐blockers	4386 (28.8)	78 323 (14.6)	0.350	4221 (28.2)	4286 (28.6)	0.010
Calcium channel blockers	1507 (9.9)	48 728 (9.1)	0.028	1486 (9.9)	1573 (10.5)	0.019
Antiarrhythmics	7163 (47.0)	90 282 (16.8)	0.685	6904 (46.1)	6880 (46.0)	0.003
Lipid modifying agents	3053 (20.0)	104 971 (19.5)	0.013	3016 (20.1)	3178 (21.2)	0.027

Abbreviations: ACE, angiotensin converting enzyme; ALT, alanine aminotransferase; AST, aspartate aminotransferase; BMI, body mass index; CKD, chronic kidney disease; HbA_1C_, haemoglobin A_1C_; HDL, high‐density lipoprotein; IHD, ischemic heart disease; *n*, number; LDL, low density lipoprotein, MBS, metabolic bariatric surgery; PSM, propensity score matching, SMD, standardized mean difference; SD, standard deviation; T2D, type 2 diabetes.

^a^ Includes: metformin, glipizide, sitagliptin, glimepiride, pioglitazone, glyburide, empagliflozin, dapagliflozin, acarbose, miglitol, canagliflozin, linagliptin, saxagliptin, alogliptin, ertugliflozin, chlorpropamide, nateglinide, repaglinide, rosiglitazone.

^b^ Includes: liraglutide, semaglutide, dulaglutide, tirzepatide, exenatide.

**TABLE 2:** Associations of MBS with major adverse hepatic and extrahepatic clinical outcomes and all‐cause mortality in individuals living with SLD.

Outcome	Number of patients in MBS	Patients with outcome in MBS, No. (%)	Number of patients in no‐MBS	Patients with outcome in no‐MBS, No. (%)	Hazard ratio (CI)
Hepatic outcomes					
MALO	14 970	499 (3.3)	14 968	590 (3.9)	0.84 (0.75–0.95)
Cirrhosis	14 970	223 (1.5)	14 969	259 (1.7)	0.86 (0.72–1.02)
Decompensated cirrhosis	14 970	309 (2.1)	14 969	375 (2.5)	0.82 (0.70–0.95)
HCC	14 970	≤10^a^ (0.1)	14 970	17 (0.1)	0.47 (0.20–1.08)
Intrahepatic cancer^b^	14 961	11 (0.1)	14 953	29 (0.2)	0.38 (0.19–0.75)
Liver transplant	14 970	≤10^a^ (0.1)	14 970	13 (0.1)	0.62 (0.26–1.48)
Cardiovascular outcomes					
MACE	13 799	674 (4.9)	13 450	1237 (9.2)	0.52 (0.47–0.57)
Acute MI	14 765	193 (1.3)	14 655	327 (2.2)	0.58 (0.49–0.69)
Stroke	14 637	353 (2.4)	14 529	527 (3.6)	0.66 (0.58–0.75)
Heart failure	14 197	395 (2.8)	13 953	854 (6.1)	0.45 (0.40–0.50)
Renal outcomes					
MAKE	14 904	77 (0.5)	14 838	140 (0.9)	0.54 (0.41–0.72)
CKD5	14 950	25 (0.2)	14 938	47 (0.3)	0.53 (0.33–0.86)
ESRF	14 911	54 (0.4)	14 870	98 (0.7)	0.55 (0.39–0.76)
Dialysis	14 928	57 (0.4)	14 876	107 (0.7)	0.53 (0.38–0.73)
Obesity‐related cancers					
Composite	14 422	290 (2.0)	13 857	472 (3.4)	0.58 (0.50–0.67)
Colorectal	14 914	33 (0.2)	14 831	70 (0.5)	0.47 (0.31–0.71)
Breast	14 770	99 (0.7)	14 511	179 (1.2)	0.54 (0.42–0.69)
Uterine	14 904	44 (0.3)	14 782	73 (0.5)	0.59 (0.41–0.86)
Ovarian	14 940	25 (0.2)	14 911	37 (0.2)	0.67 (0.40–1.11)
Renal	14 906	32 (0.2)	14 873	63 (0.4)	0.50 (0.33–0.77)
Pancreatic	14 943	22 (0.1)	14 919	35 (0.2)	0.62 (0.37–1.06)
Gastric	14 929	11 (0.1)	14 954	12 (0.1)	0.91 (0.40–2.06)
Oesophagus	14 964	≤10^a^ (0.1)	14 954	11 (0.1)	0.90 (0.38–2.12)
Gallbladder	14 970	0 (0.0)	14 964	≤10^a^ (0.1)	n/a
Thyroid	14 889	45 (0.3)	14 856	49 (0.3)	0.91 (0.61–1.36)
Meningeal	14 969	≤10^a^ (0.1)	14 964	≤10^a^ (0.1)	0.20 (0.02–1.69)
Multiple myeloma	14 957	11 (0.1)	14 935	26 (0.2)	0.42 (0.21–0.85)
All‐cause mortality	14 970	327 (2.2)	14 969	665 (4.4)	0.49 (0.43–0.56)

Abbreviations: CI, confidence interval; CKD5, chronic kidney disease stage 5; ESRF, end stage renal failure; HCC, hepatocellular carcinoma; MACE, major adverse cardiovascular outcomes; MAKE, major adverse kidney events; MALO, major adverse liver outcomes; MBS, metabolic bariatric surgery; MI, myocardial infarction; SLD, steatotic liver disease.

^a^ To protect patients' anonymity on TriNetX, if the sample size is ≤10, the exact number of patients is concealed.

^b^ Intrahepatic cancer includes hepatocellular carcinoma and intrahepatic biliary tree cancers; intrahepatic cancer is a composite of MALO.

### 3.2 Patient characteristics

Baseline characteristics are summarized in Table 1. The mean age after PSM in MBS cohort was 46.7 (±12.2) and 47.4 years (±13.9) in no‐MBS cohort. 74.3% and 75.7% of all subjects were female in the MBS and no‐MBS cohorts respectively. Overall, after PSM, the groups were considered well‐balanced (Table 1 and Figure S1). The mean follow‐up was 4.1 years in both cohorts. Baseline characteristics before and after PSM for all subgroup analyses are presented in Tables S11–S18.

### 3.3 Primary outcome measure: major adverse liver outcomes

**FIGURE 2:**
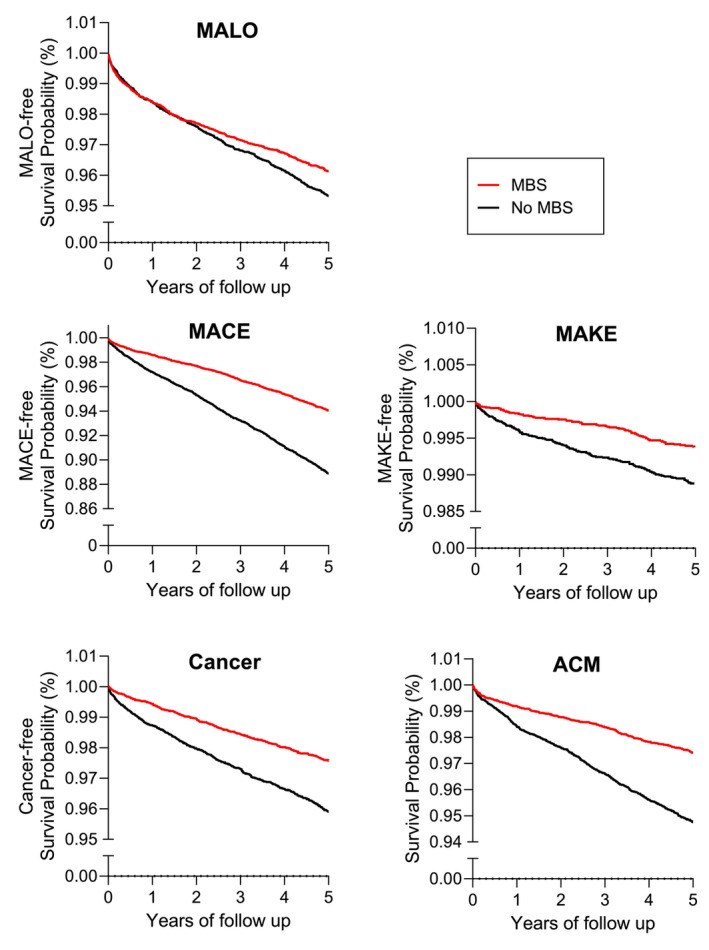
Event‐free survival probabilities in patients with SLD and history of metabolic bariatric surgery as compared to the non‐surgical SLD cohort. ACM, all‐cause mortality; CANCER, obesity‐related cancers; CI, confidence interval; MACE, major adverse cardiovascular outcomes; MAKE, major adverse kidney events; MALO, major adverse liver outcomes; MBS, metabolic bariatric surgery; SLD, steatotic liver disease.

**FIGURE 3:**
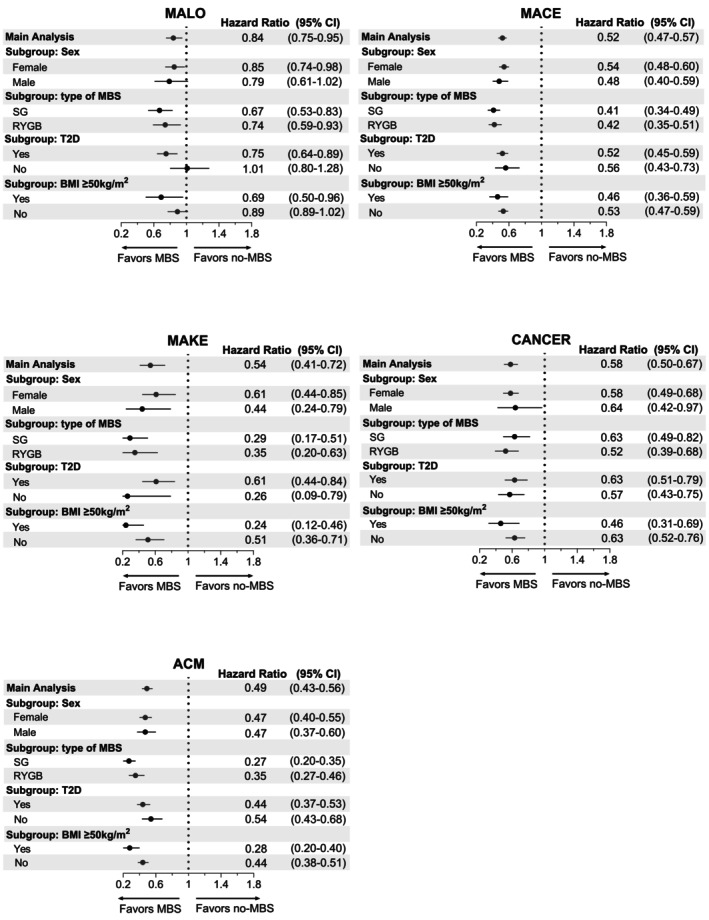
Hazard ratios of adverse hepatic/extrahepatic outcomes and all‐cause mortality in MBS and no‐MBS cohort with subgroup analyses for sex, type of MBS, and presence or absence of additional metabolic risk factors (T2D and BMI ≥50 kg/m^2^). ACM, all‐cause mortality; BMI, body mass index; CANCER, obesity‐related cancers; CI, confidence interval; MACE, major adverse cardiovascular outcomes; MAKE, major adverse kidney events; MALO, major adverse liver outcomes; MBS, metabolic bariatric surgery; RYGB, Roux‐en‐Y gastric bypass; SG, sleeve gastrectomy; T2D, type 2 diabetes.

*MALO*: The HR of MALO in the primary analysis was 0.84 (0.75–0.95), favouring those in the MBS cohort (Table 2, Figure 2). The reduced HR of MALO in the MBS cohort was confirmed in subgroup analyses according to the type of surgery (SG: 0.67 (0.53–0.83); RYGB: 0.74 (0.59–0.93)), presence of metabolic risk factors (T2D: 0.75 (0.64–0.89), BMI ≥50 kg/m^2^: 0.69 (0.50–0.96)) and in female patients (0.85 (0.74–0.98)) (Figure 3, Tables S19–26 and Figures S2–S5). In male patients and those without additional metabolic risk factors, the HR did not reach statistical significance (Males: 0.79 (0.61–1.02), No‐T2D: 1.01 (0.80–1.28); BMI < 50 kg/m^2^: 0.89 (0.77–1.02), Figure 3, Tables S19–26 and Figures S2–S5).

*Individual MALO components*: This was only significant for decompensated cirrhosis (0.82 (0.70–0.95), Table 2). For other components (cirrhosis, HCC, liver transplant), there was a non‐significant trend toward a reduced HR in the MBS cohort (Table 2). Of note, the HR in HCC alone did not meet the threshold for statistical significance (0.47 (0.20–1.08)) but the HR of intrahepatic cancers (including both HCC and intrahepatic biliary tract cancers) was reduced in the MBS cohort (0.38 (0.19–0.75), Table 2). In subgroup analysis, patients with both SLD and T2D had a reduced rate of all individual components of MALO except liver transplant, with a strong reduction in HR of HCC (0.09 (0.02–0.38), Table S21).

### 3.4 Secondary outcomes measures: major adverse extrahepatic outcomes and all‐cause mortality

*MACE*: There was an unequivocal reduction in HR of MACE and all its individual components (acute myocardial infarction, stroke, and heart failure) in the secondary analysis (MACE 0.52 (0.47–0.57); Table 2, Figure 2) and all subgroup analyses except the HR of acute MI in non‐diabetic patients, which did not reach statistical significance (0.71 (0.47–1.06); Figure 3, Tables S19–S26 and Figures S2–S5).

*MAKE*: The HR of MAKE was 0.54 (0.41–0.72) (Table 2, Figure 2) and the reduced HR was noted in all subgroup analyses (Figure 3, Tables S19–S26 and Figures S2‐5). Reduced HR of end‐stage renal failure and progression to dialysis was significant in all subgroups (Tables S19–S26) except the nondiabetic patients (Table S22). Reduced HR of chronic kidney disease 5 was significant in males (Table S26), patients with a history of SG (Table S21), and BMI ≥50 kg/m^2^ (Table S23).

*Obesity‐associated cancers*: The HR of obesity‐related cancers was 0.58 (0.50–0.67) (Table 2, Figure 2) and similarly to previous composite outcomes, the reduced HR was confirmed in all subgroup analyses (Figure 3, Tables S19–S26 and Figures S2–S5).

*Individual obesity‐associated cancers*: Across the whole cohort, significant benefit from MBS was noted for cancers of colon and rectum (0.47 (0.31–0.70)), breast (0.54 (0.42–0.69)), uterus (0.59 (0.41–0.86)), kidney (0.50 (0.33–0.77)), and multiple myeloma (0.42 (0.21–0.85), Table 2). In addition, female patients had reduced HR of ovarian (0.56 (0.33–0.94)) and pancreatic (0.48 (0.26–0.89)) cancers (Table S25).

*ACM*: The HR of ACM was 0.49 (0.43–0.56) (Table 2, Figure 2). The reduced HR of ACM was confirmed in all subgroup analyses (Figure 3, Tables S19–S26 and Figures S2–S5).

### 3.5 Postoperative complications

The overall risk of any complication within 30 days of MBS was 1.5% (Table S27). Individual risks of leak or superficial wound infection were both 0.1%, risks of venous thromboembolism or bleeding were 0.5% and 30‐day mortality was 0.2%.

## 4 DISCUSSION

In this real‐world retrospective analysis of data from individuals with SLD (consisting of MASLD and MetALD with hepatic steatosis and/or steatohepatitis) with a mean follow‐up of 4.1 years, we demonstrate that MBS is associated with significantly reduced rates of all major composite outcomes including hepatic and extra‐hepatic complications. Individuals with a history of MBS had a 16% reduction in the hazard of MALO, 48% in MACE, 46% in MAKE, 42% in obesity‐associated cancer, and 51% in ACM. The associations of MBS with improved clinical outcomes are particularly noticeable in patients with additional metabolic risk factors such as T2D or severe obesity. In patients without these risk factors, MBS was still associated with lower rates of major adverse extrahepatic outcomes, but there was no significant benefit on adverse hepatic events in these subgroups.

### 4.1 Benefits of MBS on hepatic clinical outcomes

In a study of 1158 patients with a histological diagnosis of steatohepatitis and fibrosis, Aminian et al. report an adjusted HR of MALO of 0.12 after MBS as compared to the non‐surgical cohort.^13^ In our analysis, the HR of MALO was much higher at 0.84 (0.75–0.95). This discrepancy likely relates to differences in inclusion criteria and population demographics; we included a broader clinical population, comprising patients with simple steatosis and steatohepatitis in the context of either MASLD or MetALD, whereas Aminian et al. focus on patients with more advanced stages of MASLD, that is, steatohepatitis and fibrosis, which are associated with a much higher risk of adverse liver‐related outcomes.^16^ Moreover, Aminian et al. restrict their analysis to SG and RYGB only; we additionally included patients with other, less effective types of MBS, such as laparoscopic adjustable gastric band LAGB.^22^ Hence, given the expected lower risk of adverse hepatic outcomes in patients with early stages of SLD and a broader definition of MBS in our analysis, a more modest benefit of surgery is not surprising. A subgroup analysis focusing specifically on patients with liver fibrosis would allow for further comparisons; however, it was not feasible due to a very low number of patients with the respective ICD‐10 code. However, the subgroup analyses focusing individually on SG and RYGB confirm that both of these procedures are associated with improved liver outcomes in patients with SLD.

The positive association between MBS and reduction in MALO in patients with SLD was recently confirmed in a large meta‐analysis of more than 16 million MBS patients and 10 million controls, demonstrating significantly reduced HR of MALO in the surgical group (HR 0.33, 0.31–0.34).^23^ This contrasts with the findings of other authors reporting no significant risk reduction of adverse hepatic outcomes after MBS. Alkharaiji et al.^24^ examined high‐risk patients with advanced fibrosis and insulin‐treated T2D with a follow‐up of 5 years, which might be too short to demonstrate a beneficial impact of MBS, given the timeframe for fibrosis resolution after surgery.^10^ Hagstrom et al.^25^ reported on participants from the Swedish Obese Subjects Study, where VBG was the predominant bariatric procedure. VBG is no longer performed due to significant risk of long‐term complications^4^, ^26^ and for this reason, we excluded patients with a history of this procedure from our analysis.

The liver‐related morbidity and mortality increase with worsening severity of SLD.^6^ It is therefore important to highlight that in our study, MBS was associated with reduced hazard of decompensated cirrhosis, further confirmed in subgroup analyses for both SG and RYGB and in patients with T2D. The trends for reduced hazards of HCC and liver transplant in the MBS cohort did not reach statistical significance, likely due to low event rate in both cohorts (0.1%) and insufficient follow‐up time (4.1 years). Longer follow‐up studies may further reveal the protective effect of MBS on advanced liver disease.

### 4.2 Benefits of MBS on extrahepatic clinical outcomes and ACM

Several observational studies reported reduced cardiovascular risk in patients with SLD after MBS.^13^, ^27^, ^28^, ^29^, ^30^, ^31^ A recent TriNetX‐based analysis identified significantly lower HR of heart failure (0.60 (0.51–0.70)), cerebrovascular events (0.59 (0.51–0.69)), and ACM (HR 0.56 (0.42–0.74)) in this group of patients.^28^ Reduced risk of cardiovascular events in patients with SLD after MBS was further confirmed in a recent meta‐analysis.^32^ In our study, the observed risk reduction in cardiovascular morbidity and ACM was very similar (heart failure: 0.48 (0.40–0.50), stroke: 0.66 (0.58–0.75), and ACM: 0.49 (0.43–0.56)).

With respect to renal outcomes, a number of observational studies report the association of MBS with either improved estimated glomerular filtration rate and albuminuria,^33^, ^34^, ^35^ or lower risk of decline in renal function.^36^ In our study, MBS was associated with reduced HR of MAKE (0.54 (0.41–0.72)), including in all subgroup analyses, further confirming the positive association between MBS and renal outcomes.

With respect to cancer outcomes, we found a reduced HR of obesity‐related cancers (0.58 (0.50–0.67)) and the beneficial effects of MBS were seen individually in cancers of the colon and rectum, breast, uterus, kidney, and multiple myeloma. Rustigi et al. performed a large retrospective analysis of patients with severe obesity and SLD and found that among those who had MBS, the risk of obesity‐related cancer was reduced by 25% as compared to those without MBS.^37^ There was significant risk reduction in colorectal, pancreatic, endometrial, and thyroid cancers as well as HCC and multiple myeloma.^37^ Our study further confirms the beneficial effect of MBS on selected obesity‐related cancers in patients with SLD.

Finally, for ACM, evidence from observational studies suggests 24%–55% lower risk of ACM in patients with obesity undergoing MBS.^29^, ^38^, ^39^, ^40^ Therefore, the ACM HR of 0.49 (0.43–0.56) in SLD patients after MBS in our study is consistent. The main drivers for decreased mortality after MBS are reductions in coronary artery disease‐, diabetes‐, and cancer‐specific mortality,^38^ although it is important to note that historically, MBS, and especially RYGB, was associated with increased mortality from accidental death and suicide.^38^ Data on specific causes of death is not provided via the TrinetX platform; therefore, we could not examine the rates of accidental and suicide death in our cohorts. With regard to postoperative mortality, the 30‐day risk observed in this study fell within the generally accepted range of 0.04%–0.2%,^4^ reflecting the notion that MBS is considered safe in patients with early‐stage SLD. In fact, the role for MBS in the management of patients with non‐cirrhotic SLD has been recognized in clinical guidelines on indications for MBS and the management of SLD, which recommend consideration of MBS in patients with SLD and co‐existing obesity.^4^, ^5^

### 4.3 Mechanisms of MBS in SLD

Even moderate weight reduction is associated with SLD improvement.^41^ Therefore, SLD regression after MBS is expected, given the significant and durable surgically induced weight loss. However, in addition to weight‐dependent effects, human and animal studies suggest that MBS induces metabolic changes independently of weight loss. For example, improved hepatic insulin sensitivity and high rates of T2D medication discontinuation occur within days of surgery and before any significant weight loss.^42^, ^43^ SLD shares several key metabolic features with T2D, including insulin resistance, hyperglycemia, and chronic low‐grade inflammation^44^; therefore, it is possible that post‐surgical improvements in SLD are closely related to the anti‐T2D mechanisms of MBS. Finally, in addition to weight‐dependent and glucoregulatory effects of MBS, rodent models revealed its direct impact on hepatic lipid metabolism.^45^, ^46^ Some of the mediators of the metabolic response after MBS include gastrointestinal hormones, bile acids, adipokines, microbiome, and immune cells^47^, ^48^, ^49^ but the exact molecular mechanisms remain the subject of ongoing research.

### 4.4 MBS in context of emerging pharmacotherapies in SLD

In recent years, there have been several pharmacological agents showing promising results in SLD^50^, ^51^ with glucagon‐like peptide 1 receptor agonists (GLP‐1RA) revolutionizing the management of obesity and T2D and consequently changing the trends in MBS utilization.^52^ Whilst MBS results in increased production of GLP‐1,^47^ GLP‐1RA potentiate its physiological effects. These include increased insulin secretion, intestinal transit regulation, and induction of satiety, leading to weight loss.^53^ Whether GLP‐1RA have a direct effect in the liver remains to be determined.^53^ MBS still confers superior benefits on weight reduction,^54^ but it is unknown how the impact of GLP‐1RA on clinical outcomes in SLD compares to the effects of MBS. It is likely that both medical and surgical options will co‐exist in the armamentarium of weight management centres, allowing for more personalized treatment plans and various combinations of medical and surgical options into a multi‐step approach to treat obesity and its associated co‐morbidities, including SLD.

### 4.5 Study limitations

The observational nature of this study precludes any causal inference. Furthermore, some of the data might be incomplete or inaccurate, reflecting errors in coding or underdiagnosis of SLD. Therefore, the results should be viewed as an approximation of the true association between MBS and clinical outcomes in SLD.

A universal limitation of the TriNetX platform is the absence of patient‐level data, due to the protection of patients' anonymity, with several potential consequences for each study; this is counterbalanced by the sheer volume of patient outcomes. With respect to this specific analysis, individual‐level data on SLD severity, degree of hepatic fibrosis, alcohol intake, and socioeconomic status would facilitate any potential baseline differences between the cohorts to be statistically accounted for. Potential baseline differences in these characteristics may independently impact the outcomes. Additionally, due to the concealment of exact dates of diagnoses, we are unable to estimate the lead‐time bias in this study, which could have affected either cohort. For example, patients in the MBS cohort may have been diagnosed with SLD at an earlier stage (than the no‐MBS cohort) during preoperative medical evaluation. This represents not only detection bias, but also lead‐time bias, because their time‐to‐event appears longer. Conversely, patients in the no‐MBS cohort also could have been diagnosed in the early disease stage, for example, via routine testing or evaluation for another clinical indication. In such cases, they might appear to have longer time‐to‐event as compared to the MBS cohort whose follow‐up started only after their surgery. Overall, we cannot exclude that lead‐time bias affected our results. Unfortunately, we cannot estimate the degree or directionality of its impact due to the unavailability of patient‐level data.

The applicability of our results might be limited by possible selection bias from large academic centres, which are more likely to contribute to TriNetX; and by including in our analysis patients with a history of procedures like LAGB, which are nowadays performed much less commonly.^55^ Furthermore, it is possible that patients in the MBS cohort have received more follow‐up and enhanced preventative measures than the patients in the no‐MBS cohort, thus driving better clinical outcomes in the former cohort.^56^, ^57^

Finally, patients with SLD‐associated fibrosis are likely underrepresented, and patients with cirrhosis have been excluded altogether; therefore, this study does not provide sufficient evidence to assess the associations between MBS and clinical outcomes in patients with late stages of SLD. Given the link between fibrosis and cirrhosis with increased hepatic morbidity and mortality, and the ongoing debate on the safety of MBS in cirrhosis, this remains an important area for future research.

## 5 CONCLUSION

In conclusion, we demonstrate an association of MBS with a significant reduction in the rate of major adverse hepatic and extrahepatic clinical outcomes and lower ACM in a large cohort of patients with SLD, with benefits confirmed in individuals with significant metabolic risk factors (T2D and BMI ≥50 kg/m^2^) and those with a history of either SG or RYGB. A diagnosis of SLD should be considered a significant clinical criterion to prompt referrals of patients living with obesity to bariatric surgical centres.

## FUNDING INFORMATION

None.

## CONFLICT OF INTEREST STATEMENT

Eric G Sheu has research support from Novo Nordisk and NIH, intellectual property related to diabetes and obesity treatment and has received past consulting/educational support from Vicarious Surgical, Intuitive, and Cine‐Med. Uazman Alam has received honoraria from Procter & Gamble, Viatris, Eli Lilly, Grunenthal and Sanofi for educational meetings and funding for attendance at an educational meeting from Diiachi Sankyo. Uazman Alam has also received investigator‐led research funding by Procter & Gamble and is a council member of the Royal Society of Medicine's Vascular, Lipid & Metabolic Medicine Section. Daniel J Cuthbertson has received investigator‐initiated grants from Astra Zeneca and Novo Nordisk, support for education from Perspectum and Madrigal with any financial remuneration from pharmaceutical company consultation made to the University of Liverpool.

## Supporting information


**Data S1:** Supporting Information.

## Data Availability

Data used in this study was collected solely from the TriNetX network (https://trinetx.com). This data is not available publicly due to privacy restrictions in place. However, accredited researchers registered with TriNetX might request permission to access data via TriNetX. This may require a data‐sharing agreement and may incur data access fees.
