# Supplementary material for: Metabolic bariatric surgery is associated with reduced adverse hepatic and extrahepatic outcomes, and lower all‐cause mortality, in patients with steatotic liver disease
Source: Diabetes Obes Metab. 2025 Sep 30;28(1):174–85. doi: 10.1111/dom.70173 (PMC12673435; doi:10.1111/dom.70173)
Supplement: Supplementary file 1 — Data S1: Supporting Information. [file DOM-28-174-s001.docx]

**Supplementary Appendix**

**Supplementary Methods**

***The TriNetX platform***

TriNetX is a global federated health research network that derives de-identified data from electronic medical records from healthcare organizations worldwide, with most providers located in North America. This analysis was conducted on the Research Network, which contains data from over 135 million patients.

***Statistical analysis***

Statistical analysis was performed *in situ* within the TriNetX platform. Baseline characteristics are presented as mean ± standard deviation (SD). Hazard ratios (HR) are presented with 95% confidence intervals (CI). PSM was performed using logistic regression. TriNetX uses greedy nearest-neighbor matching with a caliper of 0.1 pooled SD and difference between propensity scores <0.1. We assessed covariate balance between groups using the standardized mean difference (SMD). Covariates with SMD<0.1 were considered well matched. Survival analysis was performed to estimate the probability of an outcome. TriNetX uses the R Survival package V.3.2-3 to perform log-rank test and generate HR for each outcome of interest. P value of <0.05 was considered significant.

**Table S1** ICD-10-CM codes used to identify subjects with steatotic liver disease.

| **Code** | **Code Type** | **Description** |
| --- | --- | --- |
| K76.0 | ICD10CM | Fatty (change of) liver, not elsewhere classified |
| K75.81 | ICD10CM | Nonalcoholic steatohepatitis (NASH) |

**Table S2** Codes used to identify subjects with exclusion criteria.

| **Code** | **Code Type** | **Description** |
| --- | --- | --- |
| **Diseases of liver (K70-K77)** | | |
| K70 | ICD10CM | Alcoholic liver disease |
| K71 | ICD10CM | Toxic liver disease |
| K74.3 | ICD10CM | Primary biliary cirrhosis |
| K74.4 | ICD10CM | Secondary biliary cirrhosis |
| K74.5 | ICD10CM | Biliary cirrhosis, unspecified |
| K75.4 | ICD10CM | Autoimmune hepatitis |
| K76.5 | ICD10CM | Hepatic veno-occlusive disease |
| **Viral hepatitis (B15-B19)** | | |
| B15 | ICD10CM | Acute hepatitis A |
| B16 | ICD10CM | Acute hepatitis B |
| B17 | ICD10CM | Other acute viral hepatitis |
| B18 | ICD10CM | Chronic viral hepatitis |
| B19 | ICD10CM | Unspecified viral hepatitis |
| **Disorders of gallbladder, biliary tract and pancreas (K80-K87)** | | |
| K83.01 | ICD10CM | Primary sclerosing cholangitis |
| **Diseases of veins, lymphatic vessels and lymph nodes, not elsewhere classified (I80-I89)** | | |
| I82.0 | ICD10CM | Budd-Chiari syndrome |
| **Endocrine, nutritional and metabolic diseases (E00-E89)** | | |
| E88.01 | ICD10CM | Alpha-1-antitrypsin deficiency |
| E83.01 | ICD10CM | Wilson's disease |
| E83.11 | ICD10CM | Hemochromatosis |
| E10 | ICD10CM | Type 1 diabetes mellitus |
| **Other** | | |
| 43842 | CPT | Gastric restrictive procedure, without gastric bypass, for morbid obesity; vertical-banded gastroplasty |

**Table S3** Codes used to identify subjects with the history of metabolic bariatric surgery in primary and secondary analysis.

| **Code** | **Code Type** | **Description** |
| --- | --- | --- |
| **Sleeve gastrectomy** | | |
| 43775 | CPT | Laparoscopy, surgical, gastric restrictive procedure; longitudinal gastrectomy (ie, sleeve gastrectomy) |
| 87604009 | SNOMED | Sleeve resection of stomach |
| 427074001 | SNOMED | Laparoscopic sleeve gastrectomy |
| **Gastric bypass** | | |
| 43645 | CPT | Laparoscopy, surgical, gastric restrictive procedure; with gastric bypass and small intestine reconstruction to limit absorption |
| 43847 | CPT | Gastric restrictive procedure, with gastric bypass for morbid obesity; with small intestine reconstruction to limit absorption |
| 43846 | CPT | Gastric restrictive procedure, with gastric bypass for morbid obesity; with short limb (150 cm or less) Roux-en-Y gastroenterostomy |
| 43644 | CPT | Laparoscopy, surgical, gastric restrictive procedure; with gastric bypass and Roux-en-Y gastroenterostomy (roux limb 150 cm or less) |
| 1014146 | CPT | Gastric restrictive procedure, with gastric bypass for morbid obesity |
| **Biliopancreatic diversion** | | |
| 43845 | CPT | Gastric restrictive procedure with partial gastrectomy, pylorus-preserving duodenoileostomy and ileoileostomy (50 to 100 cm common channel) to limit absorption (biliopancreatic diversion with duodenal switch) |
| **Adjustable Gastric Band** | | |
| Z46.51 | ICD10CM | Encounter for fitting or adjusting a gastric lap band |
| 43770 | CPT | Placement of adjustable gastric restrictive device |
| **Generic** | | |
| Z98.84 | ICD10CM | Bariatric surgery status |
| 1007385 | CPT | Bariatric Surgery Procedures |
| 1007418 | CPT | Gastric restrictive procedure, open |
| 1007387 | CPT | Laparoscopy, surgical, gastric restrictive procedure |
| 1014145 | CPT | Laparoscopy, surgical, gastric restrictive procedure |
| 1007403 | CPT | Gastric restrictive procedure, without gastric bypass, for morbid obesity |
| 43843 | CPT | Gastric restrictive procedure, without gastric bypass, for morbid obesity; other than vertical-banded gastroplasty |
| 430715008 | SNOMED | Bariatric operative procedure |

**Table S4** Codes used to identify patients with history of sleeve gastrectomy or Roux-en-Y gastric bypass in subgroup analysis.

| **Code** | | **Code Type** | **Description** |
| --- | --- | --- | --- |
| **Sleeve gastrectomy** | | | |
| Z98.84 | | ICD10CM | Bariatric surgery status |
| **AND** any one of the following: | | | |
|  | 0DB60Z3 | ICD10PCS | Excision of Stomach, Open Approach, Vertical |
|  | 0DB68Z3 | ICD10PCS | Excision of Stomach, Via Natural or Artificial Opening Endoscopic, Vertical |
|  | 0DB64Z3 | ICD10PCS | Excision of Stomach, Percutaneous Endoscopic Approach, Vertical |
|  | 0DB63Z3 | ICD10PCS | Excision of Stomach, Percutaneous Approach, Vertical |
|  | 0DB63ZZ | ICD10PCS | Excision of Stomach, Percutaneous Approach |
|  | 0DB67ZZ | ICD10PCS | Excision of Stomach, via Natural or Artificial Opening |
|  | 0DB60ZZ | ICD10PCS | Excision of Stomach, Open Approach |
| **OR** any one of the following: | | | |
| 43775 | | CPT | Laparoscopy, surgical, gastric restrictive procedure; longitudinal gastrectomy (ie, sleeve gastrectomy) |
| 43843 | | CPT | Gastric restrictive procedure, without gastric bypass, for morbid obesity, other than vertical-banded gastroplasty |
| 87604009 | | SNOMED | Sleeve resection of stomach |
| 427074001 | | SNOMED | Laparoscopic sleeve gastrectomy |
| **Roux-en-Y gastric bypass** | | | |
| Z98.84 | | ICD10CM | Bariatric surgery status |
| **AND** any one of the following: | | | |
|  | 0D160ZA | ICD10PCS | Bypass Stomach to Jejunum, Open Approach |
|  | 0D1607A | ICD10PCS | Bypass Stomach to Jejunum with Autologous Tissue Substitute, Open Approach |
|  | 0D1647A | ICD10PCS | Bypass Stomach to Jejunum with Autologous Tissue Substitute, Percutaneous Endoscopic Approach |
|  | 0D164ZA | ICD10PCS | Bypass Stomach to Jejunum, Percutaneous Endoscopic Approach |
| **OR** any one of the following: | | | |
| 43645 | | CPT | Laparoscopy, surgical, gastric restrictive procedure; with gastric bypass and small intestine reconstruction to limit absorption |
| 43847 | | CPT | Gastric restrictive procedure, with gastric bypass for morbid obesity; with small intestine reconstruction to limit absorption |
| 43846 | | CPT | Gastric restrictive procedure, with gastric bypass for morbid obesity; with short limb (150 cm or less) Roux-en-Y gastroenterostomy |
| 43644 | | CPT | Laparoscopy, surgical, gastric restrictive procedure; with gastric bypass and Roux-en-Y gastroenterostomy (roux limb 150 cm or less) |
| 1014146 | | CPT | Gastric restrictive procedure, with gastric bypass for morbid obesity |

**Table S5** Characteristics used for propensity score matching together with associated codes.

| **Code** | **Code Type** | **Characteristic** |
| --- | --- | --- |
| n/a | | Age at index event |
| n/a | | Female sex |
| n/a | | White |
| n/a | | Black or African American |
| n/a | | Asian |
| n/a | | Hispanic or Latino |
| 9083 | TNX Curated | Body mass index |
| 9037 | TNX Curated | Hemoglobin A1c total in Blood |
| 9044 | TNX Curated | Alanine aminotransferase in Serum, Plasma or Blood |
| 9047 | TNX Curated | Aspartate aminotransferase in Serum, Plasma or Blood |
| 9004 | TNX Curated | Triglyceride in Serum, Plasma or Blood |
| 9001 | TNX Curated | Cholesterol in HDL in Serum or Plasma |
| 9002 | TNX Curated | Cholesterol in LDL in Serum or Plasma |
| E11 | ICD10CM | Type 2 diabetes mellitus |
| E78 | ICD10CM | Disorders of lipoprotein metabolism and other lipidemias |
| I10-I1A | ICD10CM | Hypertensive diseases |
| I20-I25 | ICD10CM | Ischemic heart diseases |
| I60-I69 | ICD10CM | Cerebrovascular diseases |
| F17 | ICD10CM | Nicotine dependence |
| N18 | ICD10CM | Chronic kidney disease |
| HS502 | VA | Oral hypoglycemic agents |
| HS501 | VA | Insulin |
| HS509 | VA | Other hypoglycemic agents |
| CV800 | VA | ACE inhibitors |
| CV805 | VA | Angiotensin II inhibitors |
| CV100 | VA | Beta-blockers |
| CV200 | VA | Calcium channel blockers |
| CV300 | VA | Antiarrhythmics |
| C10 | ATC | Lipid modifying agents |

**Table S6** Codes used to identify subjects with major adverse liver outcomes.

| **Code** | **Code Type** | **Description** |
| --- | --- | --- |
| **Cirrhosis** | | |
| K74.6 | ICD10CM | Other and unspecified cirrhosis of liver |
| **Decompensated cirrhosis and its complications** | | |
| I85.01 | ICD10CM | Esophageal varices with bleeding |
| I85.11 | ICD10CM | Secondary esophageal varices with bleeding |
| K72 | ICD10CM | Hepatic failure, not elsewhere classified |
| K76.82 | ICD10CM | Hepatic encephalopathy |
| R18 | ICD10CM | Ascites |
| K76.7 | ICD10CM | Hepatorenal syndrome |
| K76.81 | ICD10CM | Hepatopulmonary syndrome |
| **Hepatocellular carcinoma** | | |
| C22.0 | ICD10CM | Liver cell carcinoma |
| **Liver transplant** | | |
| Z94.4 | ICD10CM | Liver transplant status |
| 1007811 | CPT | Liver Transplantation Procedures |
| 47135 | CPT | Liver allotransplantation, orthotopic, partial or whole, from cadaver or living donor, any age |
| 174425003 | SNOMED | Orthotopic liver transplant |
| 18027006 | SNOMED | Transplantation of liver |

**Table S7** Codes used to identify subjects with major adverse cardiovascular events.

| **Code** | **Code type** | **Description** |
| --- | --- | --- |
| **Acute myocardial infarction** | | |
| I21 | ICD10CM | Acute myocardial infarction |
| I22 | ICD10CM | Subsequent ST elevation (STEMI) and non-ST elevation (NSTEMI) myocardial infarction |
| I23 | ICD10CM | Certain current complications following ST elevation (STEMI) and non-ST elevation (NSTEMI) myocardial infarction (within the 28 day period) |
| **Stroke** | | |
| I61 | ICD10CM | Nontraumatic intracerebral hemorrhage |
| I63 | ICD10CM | Cerebral infarction |
| I65 | ICD10CM | Occlusion and stenosis of precerebral arteries, not resulting in cerebral infarction |
| I66 | ICD10CM | Occlusion and stenosis of cerebral arteries, not resulting in cerebral infarction |
| **Heart failure** | | |
| I50 | ICD10CM | Heart failure |
| I11.0 | ICD10CM | Hypertensive heart disease with heart failure |

**Table S8** Codes used to identify subjects with major adverse kidney events.

| **Code** | **Code type** | **Description** |
| --- | --- | --- |
| **Stage 5 chronic kidney disease** | | |
| N18.5 | ICD10CM | Chronic kidney disease, stage 5 |
| **End stage renal disease** | | |
| N18.6 | ICD10CM | End stage renal disease |
| **Dialysis** | | |
| Z99.2 | ICD10CM | Dependence on renal dialysis |
| 1012740 | CPT | Dialysis Services and Procedures |
| 1029674 | CPT | Dialysis Circuit Procedures |
| 108241001 | SNOMED | Dialysis procedure |
| 385971003 | SNOMED | Dialysis care |

**Table S9** Codes used to identify subjects with obesity-associated cancers.

| **Code** | **Code type** | **Description** |
| --- | --- | --- |
| C22.0 | ICD10CM | Liver cell carcinoma |
| C22 | ICD10 CM | Malignant neoplasm of liver and intrahepatic bile ducts |
| C18 | ICD10CM | Malignant neoplasm of colon |
| C25 | ICD10CM | Malignant neoplasm of pancreas |
| C20 | ICD10CM | Malignant neoplasm of rectum |
| C16 | ICD10CM | Malignant neoplasm of stomach |
| C23 | ICD10CM | Malignant neoplasm of gallbladder |
| C50 | ICD10CM | Malignant neoplasm of breast |
| C54 | ICD10CM | Malignant neoplasm of corpus uteri |
| C54.1 | ICD10CM | Malignant neoplasm of endometrium |
| C15 | ICD10CM | Malignant neoplasm of esophagus |
| C64 | ICD10CM | Malignant neoplasm of kidney, except renal pelvis |
| C56 | ICD10CM | Malignant neoplasm of ovary |
| C19 | ICD10CM | Malignant neoplasm of rectosigmoid junction |
| C73 | ICD10CM | Malignant neoplasm of thyroid gland |
| C70 | ICD10CM | Malignant neoplasm of meninges |
| C90.0 | ICD10CM | Multiple myeloma |

**Table S10** Codes used to identify subjects within MBS cohort with 30-day postoperative complications.

| **Code** | **Code type** | **Description** |
| --- | --- | --- |
| K95 | ICD10CM | Complications of bariatric procedures |
| [K91.840](https://www.google.com/search?client=firefox-b-1-d&cs=0&sca_esv=cc1cc114a48836b1&sxsrf=AE3TifNIm5wl_3v_D4tS-s597pzsXQJE8Q%3A1750632887327&q=K91.840&sa=X&ved=2ahUKEwjVnNjaj4aOAxXajYkEHVNuBW0QxccNegQIExAB&mstk=AUtExfAD-eX04hbsRsiXKHIASZb8HZvjcAlzit6DuoOOqN9Z0RCpOMjQqel4stamoUoltMsCo0C8afIrWoTRUHIJ1H3tcQyT4N9uLhd8Cr2t8LLZINSAWXhbbyjuCklR2E2nectfFWUNnqtmULuyVHmE8sqrdpp0PcrVHla32rGm63oDI-Y&csui=3) | ICD10CM | Postprocedural hemorrhage of a digestive system organ or structure following a digestive system procedure |
| T81.320 | ICD10CM | Disruption or dehiscence of gastrointestinal tract anastomosis, repair, or closure |
| K95.81 | ICD10CM | Infection due to other bariatric procedure |
| T81.41 | ICD10CM | Infection following a procedure, superficial incisional surgical site |
| I82.40 | ICD10CM | Acute embolism and thrombosis of unspecified deep veins of lower extremity |

**
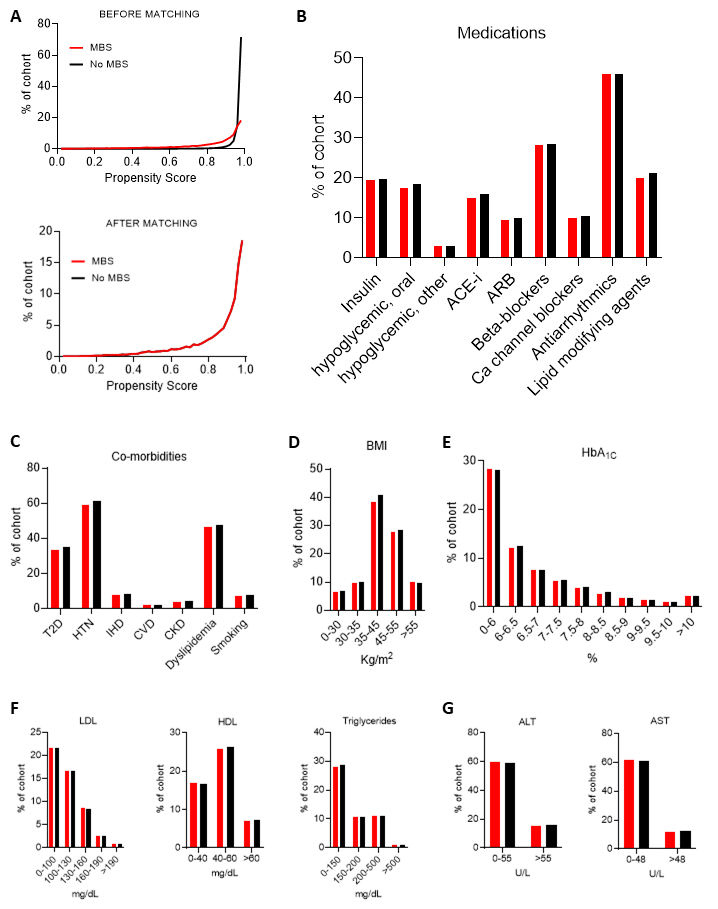
**

**Figure S1. (A)** Propensity score density function plots before (top) and after (bottom) matching for selected characteristics. **(B-G)** Distribution of selected baseline characteristics within MBS and no-MBS cohorts, medication (B), co-morbidities (C), BMI (D), HbA_1c_ (E), lipid profile (F), liver aminotransferases (G). **Abbreviations:** ACE-I, angiotensin converting enzyme inhibitor; ARB, angiotensin II receptor blocker; ALT, alanine aminotransferase; AST, aspartate aminotransferase; BMI, body mass index; Ca, calcium; CKD, chronic kidney disease; CVD, cerebrovascular disease; HbA1c, hemoglobin A1C; HDL, high density lipoprotein; HTN, hypertension; IHD, ischemic heart disease; LDL, low density lipoprotein; MBS – metabolic bariatric surgery; T2D, type 2 diabetes.

**Table S11** Baseline characteristics of patients with SLD with history of sleeve gastrectomy or without history of any metabolic bariatric surgery.

| **Characteristic** | **Before PSM** | | | **After PSM** | | |
| --- | --- | --- | --- | --- | --- | --- |
|  | **MBS**  **n=5,228** | **No-MBS**  **n= 526,555** | **SMD** | **MBS n=5,200** | **No-MBS n=5,200** | **SMD** |
| Age, mean (SD), years | 44 [12] | 52 [15] | 0.583 | 44 [12] | 45 [14] | 0.057 |
| Female sex, No. (%) | 3,854 (73.7) | 269,325 (51.4) | 0.474 | 3,827 (73.6) | 3,666 (70.5) | 0.013 |
| **Race & Ethnicity, No. (%)** | | | | | | |
| Asian | 29 (0.6) | 30,145 (5.8) | 0.301 | 29 (0.6) | 28 (0.5) | 0.003 |
| Black or African American | 836 (16.0) | 41,013 (7.8) | 0.254 | 829 (15.9) | 856 (16.5) | 0.014 |
| White | 3,654 (69.9) | 355,714 (67.9) | 0.043 | 3,634 (69.9) | 3,666 (70.5) | 0.013 |
| Hispanic or Latino | 995 (19.0) | 70,448 (13.4) | 0.152 | 990 (19.0) | 957 (18.4) | 0.016 |
| **Anthropometric and biochemical measurements, mean (SD)** | | | | | | |
| BMI, kg/m^2^ | 44.3 [7.4] | 33.3 [7.5] | 1.473 | 44.2 [7.4] | 43.8 [7.6] | 0.061 |
| HbA_1C_, *%* | 6.1 [1.2] | 6.6 [1.8] | 0.324 | 6.1 [1.2] | 6.2 [1.5] | 0.073 |
| ALT, U/L | 37 [34] | 55 [81] | 0.284 | 37 [34] | 43 [109] | 0.073 |
| AST, U/L | 29 [28] | 44 [96] | 0.216 | 29 [28] | 35 [104] | 0.081 |
| Triglycerides, mg/dL | 155 [99] | 185 [220] | 0.167 | 155 [99] | 164 [220] | 0.051 |
| HDL, mg/dL | 45 [14] | 45 [17] | 0.009 | 45 [14] | 44 [16] | 0.090 |
| LDL, mg/dL | 105 [33] | 107 [39] | 0.062 | 105 [34] | 103 [35] | 0.076 |
| **Comorbidities, No. (%)** | | | | | | |
| T2D | 1,768 (33.8) | 100,325 (19.1) | 0.337 | 1,757 (33.8) | 1,855 (35.7) | 0.040 |
| Hypertension | 3,276 (62.7) | 209,329 (40.0) | 0.467 | 3,252 (62.5) | 3,397 (65.3) | 0.058 |
| IHD | 391 (7.5) | 44,117 (8.4) | 0.035 | 390 (7.5) | 415 (8.0) | 0.018 |
| Cerebrovascular disease | 71 (1.4) | 15,684 (3.0) | 0.112 | 71 (1.4) | 83 (1.6) | 0.019 |
| Dyslipidemia | 2,647 (50.6) | 183,535 (35.0) | 0.319 | 6966 (46.5) | 7101 (47.4) | 0.018 |
| Smoking | 377 (7.2) | 43,533 (8.3) | 0.041 | 375 (7.2) | 395 (7.6) | 0.015 |
| CKD | 185 (3.5) | 19,507 (3.7) | 0.010 | 182 (3.5) | 193 (3.7) | 0.011 |

**Table S11 (continued)** Baseline characteristics of patients with SLD with history of sleeve gastrectomy or without history of any metabolic bariatric surgery.

| **Characteristic** | **Before PSM** | | | | **After PSM** | | | |
| --- | --- | --- | --- | --- | --- | --- | --- | --- |
|  | **MBS**  **n=5,228** | **No-MBS**  **n= 526,555** | | **SMD** | **MBS n=5,200** | **No-MBS n=5,200** | | **SMD** |
| **Medication, No. (%)** | | | | | | | | |
| Insulin | 1,288 (24.6) | | 35,363 (6.7) | 0.507 | 1,271 (24.4) | | 1,345 (25.9) | 0.033 |
| Oral Hypoglycemic^†^ | 1,047 (20.0) | | 60,251 (11.5) | 0.236 | 1,041 (20.0) | | 1,130 (21.7) | 0.042 |
| Other Hypoglycemic^‡^ | 218 (4.2) | | 5,423 (1.0) | 0.198 | 214 (4.1) | | 227 (4.4) | 0.012 |
| ACE inhibitors | 814 (15.6) | | 60,536 (11.6) | 0.118 | 809 (15.6) | | 878 (16.9) | 0.036 |
| Angiotensin II inhibitors | 587 (11.2) | | 39,697 (7.6) | 0.125 | 584 (11.2) | | 639 (12.3) | 0.033 |
| Beta-blockers | 1,974 (37.8) | | 76,203 (14.5) | 0.548 | 1,952 (37.5) | | 2,035 (39.1) | 0.033 |
| Calcium channel blockers | 578 (11.1) | | 47,583 (9.1) | 0.066 | 577 (11.1) | | 658 (12.7) | 0.048 |
| Antiarrhythmics | 3,469 (66.4) | | 86,537 (16.5) | 1.173 | 3,441 (66.2) | | 3,481 (66.9) | 0.016 |
| Lipid modifying agents | 1,109 (21.2) | | 102,562 (19.6) | 0.041 | 1,106 (21.3) | | 1,205 (23.2) | 0.046 |

**Abbreviations:** ACE, angiotensin converting enzyme; ALT, alanine aminotransferase; AST, aspartate aminotransferase; BMI, body mass index; CKD, chronic kidney disease; HbA_1C_, hemoglobin A**_1C_;** HDL, high-density lipoprotein; IHD, ischemic heart disease; n, number; LDL, low density lipoprotein, MBS, metabolic bariatric surgery; PSM, propensity score matching, SD, standard deviation; SLD, steatotic liver disease; SMD, standardized mean difference; T2D, type 2 diabetes.

† Includes: metformin, glipizide, sitagliptin, glimepiride, pioglitazone, glyburide, empagliflozin, dapagliflozin, acarbose, miglitol, canagliflozin, linagliptin, saxagliptin, alogliptin, ertugliflozin, chlorpropamide, nateglinide, repaglinide, rosiglitazone.

‡ Includes: liraglutide, semaglutide, dulaglutide, tirzepatide, exenatide.

**Table S12** Baseline characteristics of patients with SLD with history of Roux-en-Y gastric bypass or without history of any metabolic bariatric surgery.

| **Characteristic** | **Before PSM** | | | **After PSM** | | |
| --- | --- | --- | --- | --- | --- | --- |
|  | **MBS**  **n=4,422** | **No-MBS**  **n= 544,02** | **SMD** | **MBS n=4,368** | **No-MBS n=4,368** | **SMD** |
| Age, mean (SD), years | 45 [12] | 52 [15] | 0.562 | 45 [12] | 45 [14] | 0.018 |
| Female sex, No. (%) | 3,373 (76.7) | 278,726 (51.5) | 0.546 | 3,348 (76.6) | 3,407 (78.0) | 0.032 |
| **Race & Ethnicity, No. (%)** | | | | | | |
| Asian | 60 (1.4) | 30,230 (5.6) | 0.232 | 60 (1.4) | 46 (1.1) | 0.029 |
| Black or African American | 362 (8.2) | 41,246 (7.6) | 0.023 | 362 (8.3) | 382 (8.7) | 0.016 |
| White | 3,437 (78.2) | 371,920 (68.7) | 0.216 | 3,412 (78.1) | 3,429 (78.5) | 0.009 |
| Hispanic or Latino | 676 (15.4) | 70,726 (13.1) | 0.066 | 674 (15.4) | 643 (14.7) | 0.020 |
| **Anthropometric and biochemical measurements, mean (SD)** | | | | | | |
| BMI, kg/m^2^ | 45.4 [8.0] | 33.4 [7.6] | 1.553 | 45.4 [8.0] | 44.5 [7.9] | 0.109 |
| HbA_1C_, *%* | 6.3 [1.4] | 6.6 [1.8] | 0.204 | 6.3 [1.4] | 6.5 [1.7] | 0.118 |
| ALT, U/L | 37 [32] | 55 [81] | 0.292 | 37 [32] | 41 [65] | 0.080 |
| AST, U/L | 30 [25] | 44 [95] | 0.214 | 30 [26] | 34 [62] | 0.088 |
| Triglycerides, mg/dL | 164 [118] | 186 [219] | 0.124 | 164 [118] | 167 [161] | 0.025 |
| HDL, mg/dL | 44 [13] | 45 [17] | 0.064 | 44 [13] | 43 [15] | 0.061 |
| LDL, mg/dL | 105 [34] | 108 [39] | 0.069 | 105 [34] | 105 [36] | 0.006 |
| **Comorbidities, No. (%)** | | | | | | |
| T2D | 1,700 (38.7) | 105,264 (19.4) | 0.433 | 1,684 (38.6) | 1,667 (38.2) | 0.008 |
| Hypertension | 2,822 (64.2) | 218,139 (40.3) | 0.493 | 2,803 (64.2) | 2,860 (65.5) | 0.027 |
| IHD | 300 (6.8) | 46,430 (8.6) | 0.066 | 298 (6.8) | 286 (6.5) | 0.011 |
| Cerebrovascular disease | 70 (1.6) | 16,357 (3.0) | 0.095 | 70 (1.6) | 84 (1.9) | 0.024 |
| Dyslipidemia | 2,268 (51.6) | 191,008 (35.3) | 0.334 | 2,249 (51.5) | 2,202 (50.4) | 0.022 |
| Smoking | 283 (6.4) | 46,720 (8.6) | 0.083 | 283 (6.5) | 317 (7.3) | 0.031 |
| CKD | 140 (3.2) | 20,244 (3.7) | 0.030 | 140 (3.2) | 143 (3.3) | 0.004 |

**Table S12 (continued)** Baseline characteristics of patients with SLD with history of Roux-en-Y gastric bypass or without history of any metabolic bariatric surgery.

| **Characteristic** | **Before PSM** | | | **After PSM** | | |
| --- | --- | --- | --- | --- | --- | --- |
|  | **MBS**  **n=4,422** | **No-MBS**  **n= 544,02** | **SMD** | **MBS n=4,368** | **No-MBS n=4,368** | **SMD** |
| **Medication, No. (%)** | | | | | | |
| Insulin | 1,232 (28.0) | 36,854 (6.8) | 0.583 | 1,213 (27.8) | 1,188 (27.2) | 0.013 |
| Oral Hypoglycemic^†^ | 982 (22.3) | 61,830 (11.4) | 0.295 | 975 (22.3) | 958 (21.9) | 0.009 |
| Other Hypoglycemic^‡^ | 156 (3.5) | 5,541 (1.0) | 0.170 | 154 (3.5) | 131 (3.0) | 0.030 |
| ACE inhibitors | 798 (18.2) | 62,324 (11.5) | 0.188 | 795 (18.2) | 852 (19.5) | 0.033 |
| Angiotensin II inhibitors | 419 (9.5) | 40,685 (7.5) | 0.072 | 417 (9.5) | 436 (10.0) | 0.015 |
| Beta-blockers | 1,492 (33.9) | 78,552 (14.5) | 0.466 | 1,474 (33.7) | 1,472 (33.7) | 0.001 |
| Calcium channel blockers | 429 (9.8) | 48,907 (9.0) | 0.025 | 427 (9.8) | 425 (9.7) | 0.002 |
| Antiarrhythmics | 2,599 (59.1) | 90,388 (16.7) | 0.973 | 2,572 (58.9) | 2,591 (59.3) | 0.009 |
| Lipid modifying agents | 987 (22.5) | 105,421 (19.5) | 0.073 | 981 (22.5) | 1,023 (23.4) | 0.023 |

**Abbreviations:** ACE, angiotensin converting enzyme; ALT, alanine aminotransferase; AST, aspartate aminotransferase; BMI, body mass index; CKD, chronic kidney disease; HbA_1C_, hemoglobin A**_1C_;** HDL, high-density lipoprotein; IHD, ischemic heart disease; n, number; LDL, low density lipoprotein, MBS, metabolic bariatric surgery; PSM, propensity score matching; SD, standard deviation; SLD, steatotic liver disease; SMD, standardized mean difference; T2D, type 2 diabetes.

† Includes: metformin, glipizide, sitagliptin, glimepiride, pioglitazone, glyburide, empagliflozin, dapagliflozin, acarbose, miglitol, canagliflozin, linagliptin, saxagliptin, alogliptin, ertugliflozin, chlorpropamide, nateglinide, repaglinide, rosiglitazone.

‡ Includes: liraglutide, semaglutide, dulaglutide, tirzepatide, exenatide.

**Table S13** Baseline characteristics of patients with SLD and T2D with or without history of any metabolic bariatric surgery.

| **Characteristic** | **Before PSM** | | | **After PSM** | | |
| --- | --- | --- | --- | --- | --- | --- |
|  | **MBS**  **n=5,843** | **No-MBS**  **n= 166,598** | **SMD** | **MBS n=5,757** | **No-MBS n=5,757** | **SMD** |
| Age, mean (SD), years | 50[12] | 57 [13] | 0.634 | 50 [12] | 50 [13] | 0.004 |
| Female sex, No. (%) | 4,162 (71.4) | 90,119 (54.2) | 0.360 | 4,094 (71.1) | 4,167 (72.4) | 0.028 |
| **Race & Ethnicity, No. (%)** | | | | | | |
| Asian | 56 (1.0) | 8,884 (5.3) | 0.253 | 56 (1.0) | 59 (1.0) | 0.005 |
| Black or African American | 723 (12.2) | 16,478 (9.9) | 0.079 | 717 (12.5) | 693 (12.0) | 0.013 |
| White | 4,179 (71.6) | 113,796 (68.5) | 0.070 | 4,122 (71.6) | 4,142 (71.9) | 0.008 |
| Hispanic or Latino | 924 (15.8) | 23,732 (14.3) | 0.044 | 911 (15.8) | 864 (15.0) | 0.023 |
| **Anthropometric and biochemical measurements, mean (SD)** | | | | | | |
| BMI, kg/m^2^ | 43.1 [8.6] | 35.1 [7.8] | 0.979 | 42.9 [8.6] | 42.8 [8.8] | 0.021 |
| HbA_1C_, *%* | 6.9 [1.5] | 7.3 [1.9] | 0.285 | 6.9 [1.5] | 7.1 [1.8] | 0.146 |
| ALT, U/L | 39 [40] | 51 [82] | 0.185 | 39 [41] | 41 [53] | 0.038 |
| AST, U/L | 32 [37] | 44 [122] | 0.126 | 32 [37] | 35 [51] | 0.062 |
| Triglycerides, mg/dL | 174 [124] | 210 [282] | 0.167 | 174 [125] | 184 [228] | 0.050 |
| HDL, mg/dL | 43 [14] | 42 [15] | 0.088 | 43 [14] | 42 [15] | 0.045 |
| LDL, mg/dL | 98 [36] | 98 [39] | 0.008 | 98 [36] | 97 [38] | 0.034 |
| **Comorbidities, No. (%)** | | | | | | |
| Hypertension | 4,462 (76.5) | 111,362 (67.0) | 0.212 | 4,398 (76.4) | 4,450 (77.3) | 0.021 |
| IHD | 730 (12.5) | 27,147 (16.3) | 0.109 | 724 (12.6) | 758 (13.2) | 0.018 |
| Cerebrovascular disease | 162 (2.8) | 8,867 (5.3) | 0.130 | 162 (2.8) | 166 (2.9) | 0.004 |
| Dyslipidemia | 3,736 (64.0) | 96,269 (57.9) | 0.126 | 3,675 (63.8) | 3,612 (62.7) | 0.023 |
| Smoking | 424 (7.3) | 16,458 (9.9) | 0.094 | 1066 (7.1) | 1163 (7.8) | 0.025 |
| CKD | 369 (6.3) | 14,500 (8.7) | 0.091 | 365 (6.3) | 342 (5.9) | 0.017 |

**Table S13 (continued)** Baseline characteristics of patients with SLD and T2D with or without history of any metabolic bariatric surgery.

| **Characteristic** | **Before PSM** | | | **After PSM** | | |
| --- | --- | --- | --- | --- | --- | --- |
|  | **MBS**  **n=5,843** | **No-MBS**  **n= 166,598** | **SMD** | **MBS n=5,757** | **No-MBS n=5,757** | **SMD** |
| **Medication, No. (%)** | | | | | | |
| Insulin | 2,577 (44.2) | 36,141 (21.7) | 0.492 | 2,507 (43.5) | 2,493 (43.3) | 0.005 |
| Oral Hypoglycemic^†^ | 2,350 (40.3) | 63,944 (38.5) | 0.037 | 2,320 (40.3) | 2,335 (40.6) | 0.005 |
| Other Hypoglycemic^‡^ | 405 (6.9) | 5,249 (3.2) | 0.174 | 395 (6.9) | 392 (6.8) | 0.002 |
| ACE inhibitors | 1,311 (22.5) | 35,084 (21.1) | 0.033 | 1,298 (22.5) | 1,366 (23.7) | 0.028 |
| Angiotensin II inhibitors | 777 (13.3) | 21,778 (13.1) | 0.006 | 770 (13.4) | 776 (13.5) | 0.003 |
| Beta-blockers | 2,080 (35.7) | 39,390 (23.7) | 0.264 | 2,032 (35.3) | 2,040 (35.4) | 0.003 |
| Calcium channel blockers | 798 (13.7) | 24,654 (14.8) | 0.033 | 789 (13.7) | 854 (14.8) | 0.032 |
| Antiarrhythmics | 2,869 (49.2) | 37,314 (22.4) | 0.581 | 2,795 (48.5) | 2,785 (48.4) | 0.003 |
| Lipid modifying agents | 1,897 (32.5) | 59,217 (35.6) | 0.065 | 1,880 (32.7) | 1,921 (33.4) | 0.015 |

**Abbreviations:** ACE, angiotensin converting enzyme; ALT, alanine aminotransferase; AST, aspartate aminotransferase; BMI, body mass index; CKD, chronic kidney disease; HbA_1C_, hemoglobin A**_1C_;** HDL, high-density lipoprotein; IHD, ischemic heart disease; n, number; LDL, low density lipoprotein, MBS, metabolic bariatric surgery; PSM, propensity score matching; SD, standard deviation; SLD, steatotic liver disease; SMD, standardized mean difference; T2D, type 2 diabetes.

† Includes: metformin, glipizide, sitagliptin, glimepiride, pioglitazone, glyburide, empagliflozin, dapagliflozin, acarbose, miglitol, canagliflozin, linagliptin, saxagliptin, alogliptin, ertugliflozin, chlorpropamide, nateglinide, repaglinide, rosiglitazone.

‡ Includes: liraglutide, semaglutide, dulaglutide, tirzepatide, exenatide.

**Table S14** Baseline characteristics of patients with SLD and without T2D with or without history of any metabolic bariatric surgery.

| **Characteristic** | **Before PSM** | | | **After PSM** | | |
| --- | --- | --- | --- | --- | --- | --- |
|  | **MBS**  **n=6,199** | **No-MBS**  **n= 318,224** | **SMD** | **MBS n=5,968** | **No-MBS n=5,968** | **SMD** |
| Age, mean (SD), years | 45 [12] | 49 [14] | 0.323 | 45 [12] | 46 [14] | 0.060 |
| Female sex, No. (%) | 4,682 (75.7) | 156,421 (49.5) | 0.564 | 4,498 (75.4) | 4,589 (76.9) | 0.036 |
| **Race & Ethnicity, No. (%)** | | | | | | |
| Asian | 47 (0.8) | 18,909 (6.0) | 0.292 | 47 (0.8) | 47 (0.8) | <0.001 |
| Black or African American | 622 (10.1) | 17,595 (5.6) | 0.168 | 594 (10.0) | 629 (10.5) | 0.019 |
| White | 4,569 (73.9) | 220,725 (69.8) | 0.092 | 4,424 (74.1) | 4,447 (74.5) | 0.009 |
| Hispanic or Latino | 759 (12.3) | 49,730 (15.7) | 0.099 | 742 (12.4) | 587 (9.8) | 0.083 |
| **Anthropometric and biochemical measurements, mean (SD)** | | | | | | |
| BMI, kg/m^2^ | 42.3 [9.1] | 31.9 [7.1] | 1.271 | 41.9 [9.0] | 41.0 [9.0] | 0.102 |
| HbA_1C_, *%* | 5.6 [0.7] | 5.6 [1.1] | 0.052 | 5.6 [0.7] | 5.6 [0.8] | 0.064 |
| ALT, U/L | 40 [46] | 57 [86] | 0.256 | 40 [47] | 46 [102] | 0.081 |
| AST, U/L | 33 [44] | 46 [96] | 0.163 | 34 [45] | 37 [56] | 0.063 |
| Triglycerides, mg/dL | 144 [80] | 167 [150] | 0.193 | 144 [79] | 149 [116] | 0.050 |
| HDL, mg/dL | 47 [14] | 48 [17] | 0.055 | 47 [14] | 47 [15] | 0.007 |
| LDL, mg/dL | 110 [38] | 114 [38] | 0.098 | 110 [38] | 109 [33] | 0.030 |
| **Comorbidities, No. (%)** | | | | | | |
| Hypertension | 2,961 (47.9) | 88,567 (28.0) | 0.419 | 2,808 (47.1) | 2,845 (47.7) | 0.012 |
| IHD | 271 (4.4) | 15,685 (5.0) | 0.027 | 264 (4.4) | 305 (5.1) | 0.032 |
| Cerebrovascular disease | 94 (1.5) | 6,478 (2.0) | 0.040 | 92 (1.5) | 102 (1.7) | 0.013 |
| Dyslipidemia | 2,131 (34.5) | 78,070 (24.7) | 0.216 | 2,043 (34.2) | 2,012 (33.7) | 0.011 |
| Smoking | 433 (7.0) | 25,551 (8.1) | 0.041 | 421 (7.1) | 469 (7.9) | 0.031 |
| CKD | 108 (1.7) | 5,947 (1.9) | 0.010 | 105 (1.8) | 117 (2.0) | 0.015 |

**Table S14 (continued)** Baseline characteristics of patients with SLD and without T2D with or without history of any metabolic bariatric surgery.

| **Characteristic** | **Before PSM** | | | **After PSM** | | |
| --- | --- | --- | --- | --- | --- | --- |
|  | **MBS**  **n=6,199** | **No-MBS**  **n= 318,224** | **SMD** | **MBS n=5,968** | **No-MBS n=5,968** | **SMD** |
| **Medication, No. (%)** | | | | | | |
| ACE inhibitors | 645 (10.4) | 21,133 (6.7) | 0.134 | 611 (10.2) | 620 (10.4) | 0.005 |
| Angiotensin II inhibitors | 409 (6.6) | 14,847 (4.7) | 0.083 | 389 (6.5) | 415 (7.0) | 0.017 |
| Beta-blockers | 1,440 (23.3) | 30,408 (9.6) | 0.375 | 1,342 (22.5) | 1,410 (23.6) | 0.027 |
| Calcium channel blockers | 438 (7.1) | 18,836 (6.0) | 0.046 | 422 (7.1) | 453 (7.6) | 0.020 |
| Antiarrhythmics | 2,782 (45.0) | 44,803 (14.2) | 0.718 | 2,582 (43.3) | 2,565 (43.0) | 0.006 |
| Lipid modifying agents | 730 (11.8) | 36,298 (11.5) | 0.010 | 710 (11.9) | 761 (12.8) | 0.026 |

**Abbreviations:** ACE, angiotensin converting enzyme; ALT, alanine aminotransferase; AST, aspartate aminotransferase; BMI, body mass index; CKD, chronic kidney disease; GLP1 – glucagon like peptide 1; HbA_1C_, hemoglobin A_1C_**;** HDL, high-density lipoprotein; IHD, ischemic heart disease; n, number; LDL, low density lipoprotein, MBS, metabolic bariatric surgery; PSM, propensity score matching; SD, standard deviation; SLD, steatotic liver disease; SMD, standardized mean difference; T2D, type 2 diabetes.

† Includes: metformin, glipizide, sitagliptin, glimepiride, pioglitazone, glyburide, empagliflozin, dapagliflozin, acarbose, miglitol, canagliflozin, linagliptin, saxagliptin, alogliptin, ertugliflozin, chlorpropamide, nateglinide, repaglinide, rosiglitazone.

‡ Includes: liraglutide, semaglutide, dulaglutide, tirzepatide, exenatide.

**Table S15** Baseline characteristics of patients with SLD and BMI ≥50kg/m^2^ with or without history of any metabolic bariatric surgery.

| **Characteristic** | **Before PSM** | | | **After PSM** | | |
| --- | --- | --- | --- | --- | --- | --- |
|  | **MBS**  **n=2,283** | **No-MBS**  **n= 25,740** | **SMD** | **MBS n=2,148** | **No-MBS n=2,148** | **SMD** |
| Age, mean (SD), years | 44 [11] | 48 [14] | 0.362 | 44 [11] | 44 [14] | 0.005 |
| Female sex, No. (%) | 1,607 (70.7) | 17,106 (66.5) | 0.089 | 1,513 (70.4) | 1,486 (69.2) | 0.027 |
| **Race & Ethnicity, No. (%)** | | | | | | |
| Asian | 19 (0.8) | 272 (1.1) | 0.023 | 18 (0.8) | 13 (0.6) | 0.028 |
| Black or African American | 269 (11.8) | 3,524 (13.7) | 0.056 | 258 (12.0) | 260 (12.1) | 0.003 |
| White | 1,650 (72.6) | 18,350 (71.4) | 0.026 | 1,555 (72.4) | 1,531 (71.3) | 0.025 |
| Hispanic or Latino | 400 (17.6) | 3,041 (11.8) | 0.163 | 366 (17.0) | 366 (17.0) | <0.001 |
| **Anthropometric and biochemical measurements, mean (SD)** | | | | | | |
| BMI, kg/m^2^ | 53.1 [7.3] | 49.4 [9.3] | 0.445 | 52.9 [7.3] | 53.3 [7.5] | 0.054 |
| HbA_1C_, *%* | 6.2 [1.3] | 6.8 [1.8] | 0.381 | 6.3 [1.4] | 6.4 [1.6] | 0.101 |
| ALT, U/L | 34 [30] | 49 [123] | 0.164 | 35 [31] | 38 [51] | 0.083 |
| AST, U/L | 29 [30] | 47 [221] | 0.114 | 29 [31] | 33 [52] | 0.101 |
| Triglycerides, mg/dL | 150 [85] | 172 [155] | 0.177 | 151 [87] | 149 [78] | 0.028 |
| HDL, mg/dL | 43 [12] | 43 [15] | 0.018 | 43 [12] | 42 [14] | 0.064 |
| LDL, mg/dL | 102 [31] | 102 [36] | 0.007 | 102 [31] | 102 [34] | 0.006 |
| **Comorbidities, No. (%)** | | | | | | |
| T2D | 890 (39.1) | 8,960 (34.8) | 0.089 | 830 (38.6) | 891 (41.5) | 0.058 |
| Hypertension | 1,545 (67.9) | 14,493 (56.4) | 0.240 | 8832 (59.0) | 9187 (61.4) | 0.048 |
| IHD | 156 (6.9) | 2,905 (11.3) | 0.155 | 150 (7.0) | 141 (6.6) | 0.017 |
| Cerebrovascular disease | 37 (1.6) | 943 (3.7) | 0.127 | 37 (1.7) | 41 (1.9) | 0.014 |
| Dyslipidemia | 989 (43.5) | 9,515 (37.0) | 0.133 | 911 (42.4) | 954 (44.4) | 0.040 |
| Smoking | 154 (6.8) | 2,862 (11.1) | 0.153 | 150 (7.0) | 153 (7.1) | 0.005 |
| CKD | 102 (4.5) | 1,931 (7.5) | 0.128 | 100 (4.7) | 110 (5.1) | 0.022 |

**Table S15 (continued)** Baseline characteristics of patients with SLD and BMI ≥50kg/m^2^ with or without history of any metabolic bariatric surgery.

| **Characteristic** | **Before PSM** | | | **After PSM** | | |
| --- | --- | --- | --- | --- | --- | --- |
|  | **MBS**  **n=2,283** | **No-MBS**  **n= 25,740** | **SMD** | **MBS n=2,148** | **No-MBS n=2,148** | **SMD** |
| **Medication, No. (%)** | | | | | | |
| Insulin | 523 (23.0) | 4,545 (17.7) | 0.133 | 473 (22.0) | 493 (23.0) | 0.022 |
| Oral Hypoglycemic^†^ | 446 (19.6) | 5,651 (22.0) | 0.058 | 426 (19.8) | 466 (21.7) | 0.046 |
| Other Hypoglycemic^‡^ | 80 (3.5) | 610 (2.4) | 0.068 | 69 (3.2) | 86 (4.0) | 0.042 |
| ACE inhibitors | 418 (18.4) | 4,947 (19.2) | 0.022 | 401 (18.7) | 413 (19.2) | 0.014 |
| Angiotensin II inhibitors | 226 (9.9) | 2,887 (11.2) | 0.042 | 217 (10.1) | 224 (10.4) | 0.011 |
| Beta-blockers | 764 (33.6) | 6,638 (25.8) | 0.171 | 686 (31.9) | 720 (33.5) | 0.034 |
| Calcium channel blockers | 238 (10.5) | 3,890 (15.1) | 0.140 | 231 (10.8) | 244 (11.4) | 0.019 |
| Antiarrhythmics | 1,226 (53.9) | 7,303 (28.4) | 0.537 | 1,102 (51.3) | 1,096 (51.0) | 0.006 |
| Lipid modifying agents | 410 (18.0) | 6,622 (25.8) | 0.188 | 398 (18.5) | 436 (20.3) | 0.045 |

**Abbreviations:** ACE, angiotensin converting enzyme; ALT, alanine aminotransferase; AST, aspartate aminotransferase; BMI, body mass index; CKD, chronic kidney disease; HbA_1C_, hemoglobin A**_1C_;** HDL, high-density lipoprotein; IHD, ischemic heart disease; n, number; LDL, low density lipoprotein, MBS, metabolic bariatric surgery; PSM, propensity score matching; SD, standard deviation; SLD, steatotic liver disease; SMD, standardized mean difference; T2D, type 2 diabetes.

† Includes: metformin, glipizide, sitagliptin, glimepiride, pioglitazone, glyburide, empagliflozin, dapagliflozin, acarbose, miglitol, canagliflozin, linagliptin, saxagliptin, alogliptin, ertugliflozin, chlorpropamide, nateglinide, repaglinide, rosiglitazone.

‡ Includes: liraglutide, semaglutide, dulaglutide, tirzepatide, exenatide.

**Table S16** Baseline characteristics of patients with SLD and BMI <50kg/m^2^ with or without history of any metabolic bariatric surgery.

| **Characteristic** | **Before PSM** | | | **After PSM** | | |
| --- | --- | --- | --- | --- | --- | --- |
|  | **MBS**  **n=9,145** | **No-MBS**  **n= 394,820** | **SMD** | **MBS n=9,045** | **No-MBS n=9,045** | **SMD** |
| Age, mean (SD), years | 48 [12] | 53 [14] | 0.373 | 48 [12] | 48 [14] | 0.039 |
| Female sex, No. (%) | 6,965 (76.3) | 205,725 (52.2) | 0.519 | 6,885 (76.1) | 6,920 (76.5) | 0.009 |
| **Race & Ethnicity, No. (%)** | | | | | | |
| Asian | 85 (0.9) | 21,751 (5.5) | 0.262 | 85 (0.9) | 89 (1.0) | 0.005 |
| Black or African American | 763 (8.4) | 27,510 (7.0) | 0.052 | 755 (8.3) | 775 (8.6) | 0.008 |
| White | 6,923 (75.8) | 280,098 (71.1) | 0.107 | 6,860 (75.8) | 6,902 (76.3) | 0.011 |
| Hispanic or Latino | 1,394 (15.3) | 48,990 (12.4) | 0.082 | 1,374 (15.2) | 1,284 (14.2) | 0.028 |
| **Anthropometric and biochemical measurements, mean (SD)** | | | | | | |
| BMI, kg/m^2^ | 40.1 [7.2] | 33.0 [7.0] | 1.011 | 40.1 [7.2] | 39.9 [7.3] | 0.027 |
| HbA_1C_, *%* | 6.2 [1.3] | 6.6 [1.7] | 0.267 | 6.2 [1.3] | 6.4 [1.5] | 0.113 |
| ALT, U/L | 40 [50] | 55 [103] | 0.181 | 40 [51] | 45 [97] | 0.062 |
| AST, U/L | 34 [46] | 46 [138] | 0.116 | 34 [46] | 40 [151] | 0.053 |
| Triglycerides, mg/dL | 165 [112] | 189 [215] | 0.142 | 165 [113] | 171 [134] | 0.047 |
| HDL, mg/dL | 46 [14] | 46 [16] | 0.027 | 46 [14] | 45 [14] | 0.053 |
| LDL, mg/dL | 105 [38] | 108 [39] | 0.057 | 105 [38] | 104 [35] | 0.033 |
| **Comorbidities, No. (%)** | | | | | | |
| T2D | 3,178 (34.8) | 90,374 (22.9) | 0.264 | 3,137 (34.7) | 3,256 (36.0) | 0.028 |
| Hypertension | 5,411 (59.3) | 174,524 (44.3) | 0.303 | 5,350 (59.1) | 5,479 (60.6) | 0.029 |
| IHD | 784 (8.6) | 39,773 (10.1) | 0.052 | 782 (8.6) | 811 (9.0) | 0.011 |
| Cerebrovascular disease | 214 (2.3) | 15,131 (3.8) | 0.087 | 213 (2.4) | 204 (2.3) | 0.007 |
| Dyslipidemia | 4,435 (48.6) | 149,845 (38.0) | 0.214 | 4,372 (48.3) | 4,410 (48.8) | 0.008 |
| Smoking | 683 (7.5) | 40,583 (10.3) | 0.099 | 680 (7.5) | 688 (7.6) | 0.003 |
| CKD | 339 (3.7) | 19,707 (5.0) | 0.063 | 337 (3.7) | 360 (4.0) | 0.013 |

**Table S16 (continued)** Baseline characteristics of patients with SLD and BMI <50kg/m^2^ with or without history of any metabolic bariatric surgery.

| **Characteristic** | **Before PSM** | | | **After PSM** | | |
| --- | --- | --- | --- | --- | --- | --- |
|  | **MBS**  **n=9,145** | **No-MBS**  **n= 394,820** | **SMD** | **MBS n=9,045** | **No-MBS n=9,045** | **SMD** |
| **Medication, No. (%)** | | | | | | |
| Insulin | 1,819 (19.9) | 35,078 (8.9) | 0.318 | 1,774 (19.6) | 1,816 (20.1) | 0.012 |
| Oral Hypoglycemic^†^ | 1,653 (18.1) | 53,324 (13.5) | 0.125 | 1,640 (18.1) | 1,654 (18.3) | 0.004 |
| Other Hypoglycemic^‡^ | 288 (3.2) | 4,707 (1.2) | 0.135 | 281 (3.1) | 288 (3.2) | 0.004 |
| ACE inhibitors | 1,423 (15.6) | 54,562 (13.9) | 0.049 | 1,414 (15.6) | 1,409 (15.6) | 0.002 |
| Angiotensin II inhibitors | 932 (10.2) | 35,943 (9.1) | 0.037 | 923 (10.2) | 966 (10.7) | 0.016 |
| Beta-blockers | 2,645 (29.0) | 73,892 (18.8) | 0.241 | 2,603 (28.8) | 2,617 (28.9) | 0.003 |
| Calcium channel blockers | 911 (10.0) | 44,089 (11.2) | 0.039 | 907 (10.0) | 936 (10.3) | 0.011 |
| Antiarrhythmics | 4,551 (49.9) | 88,605 (22.5) | 0.594 | 4,468 (49.4) | 4,402 (48.7) | 0.015 |
| Lipid modifying agents | 2,032 (22.3) | 93,214 (23.7) | 0.033 | 2,022 (22.4) | 2,077 (23.0) | 0.015 |

**Abbreviations:** ACE, angiotensin converting enzyme; ALT, alanine aminotransferase; AST, aspartate aminotransferase; BMI, body mass index; CKD, chronic kidney disease; HbA_1C_, hemoglobin A**_1C_;** HDL, high-density lipoprotein; IHD, ischemic heart disease; n, number; LDL, low density lipoprotein, MBS, metabolic bariatric surgery; PSM, propensity score matching; SD, standard deviation; SLD, steatotic liver disease; SMD, standardized mean difference; T2D, type 2 diabetes.

† Includes: metformin, glipizide, sitagliptin, glimepiride, pioglitazone, glyburide, empagliflozin, dapagliflozin, acarbose, miglitol, canagliflozin, linagliptin, saxagliptin, alogliptin, ertugliflozin, chlorpropamide, nateglinide, repaglinide, rosiglitazone.

‡ Includes: liraglutide, semaglutide, dulaglutide, tirzepatide, exenatide.

**Table S17** Baseline characteristics of female patients with SLD with and without history of metabolic bariatric surgery.

| **Characteristic** | **Before PSM** | | | **After PSM** | | |
| --- | --- | --- | --- | --- | --- | --- |
|  | **MBS**  **n=11,466** | **No-MBS**  **n= 279,728** | **SMD** | **MBS n=11,217** | **No-MBS n=11,217** | **SMD** |
| Age, mean (SD), years | 46 [12] | 53 [14] | 0.497 | 46 [12] | 47 [14] | 0.027 |
| **Race & Ethnicity, No. (%)** | | | | | | |
| Asian | 82 (0.7) | 14,842 (5.3) | 0.272 | 82 (0.7) | 104 (0.9) | 0.022 |
| Black or African American | 1,576 (13.8) | 25,002 (9.0) | 0.151 | 1,537 (13.7) | 1,635 (14.6) | 0.025 |
| White | 8,454 (73.9) | 199,099 (71.5) | 0.053 | 8,282 (73.8) | 8,228 (73.4) | 0.011 |
| Hispanic or Latino | 1,593 (13.9) | 42,723 (15.3) | 0.040 | 1,572 (14.0) | 1,411 (12.6) | 0.042 |
| **Anthropometric and biochemical measurements, mean (SD)** | | | | | | |
| BMI, kg/m^2^ | 42.6 [8.8] | 34.0 [8.1] | 1.015 | 42.4 [8.8] | 42.1 [9.1] | 0.033 |
| HbA_1C_, *%* | 6.2 [1.2] | 6.6 [1.7] | 0.288 | 6.2 [1.2] | 6.3 [1.6] | 0.090 |
| ALT, U/L | 36 [45] | 49 [80] | 0.193 | 37 [46] | 41 [75] | 0.077 |
| AST, U/L | 32 [47] | 43 [93] | 0.145 | 32 [48] | 36 [63] | 0.062 |
| Triglycerides, mg/dL | 153 [88] | 174 [184] | 0.147 | 153 [89] | 160 [159] | 0.058 |
| HDL, mg/dL | 47 [14] | 48 [18] | 0.079 | 47 [14] | 46 [16] | 0.055 |
| LDL, mg/dL | 106 [36] | 110 [39] | 0.102 | 107 [36] | 106 [35] | 0.033 |
| **Comorbidities, No. (%)** | | | | | | |
| T2D | 3,628 (31.7) | 56,567 (20.3) | 0.262 | 3,511 (31.3) | 3,692 (32.9) | 0.035 |
| Hypertension | 6,383 (55.8) | 108,463 (39.0) | 0.341 | 6,211 (55.4) | 6,361 (56.7) | 0.027 |
| IHD | 721 (6.3) | 17,527 (6.3) | <0.001 | 712 (6.3) | 738 (6.6) | 0.009 |
| Cerebrovascular disease | 222 (1.9) | 7,908 (2.8) | 0.059 | 221 (2.0) | 232 (2.1) | 0.007 |
| Dyslipidemia | 5,054 (44.2) | 92,194 (33.1) | 0.228 | 4,912 (43.8) | 4,901 (43.7) | 0.002 |
| Smoking | 784 (6.9) | 21,510 (7.7) | 0.034 | 774 (6.9) | 896 (8.0) | 0.041 |
| CKD | 349 (3.0) | 8,988 (3.2) | 0.010 | 343 (3.1) | 383 (3.4) | 0.020 |

**Table S17 (continued)** Baseline characteristics of female patients with SLD with and without history of metabolic bariatric surgery.

| **Characteristic** | **Before PSM** | | | **After PSM** | | |
| --- | --- | --- | --- | --- | --- | --- |
|  | **MBS**  **n=11,466** | **No-MBS**  **n= 279,728** | **SMD** | **MBS n=11,217** | **No-MBS n=11,217** | **SMD** |
| **Medication, No. (%)** | | | | | | |
| Insulin | 2,163 (18.9) | 19,489 (7.0) | 0.360 | 2,039 (18.2) | 2,108 (18.8) | 0.016 |
| Oral Hypoglycemic^†^ | 1,983 (17.3) | 34,936 (12.6) | 0.134 | 1,930 (17.2) | 2,046 (18.2) | 0.027 |
| Other Hypoglycemic^‡^ | 327 (2.9) | 3,343 (1.2) | 0.118 | 310 (2.8) | 305 (2.7) | 0.003 |
| ACE inhibitors | 1,510 (13.2) | 28,773 (10.3) | 0.089 | 1,479 (13.2) | 1,521 (13.6) | 0.011 |
| Angiotensin II inhibitors | 1,010 (8.8) | 21,437 (7.7) | 0.041 | 985 (8.8) | 970 (8.6) | 0.005 |
| Beta-blockers | 3,071 (26.8) | 40,324 (14.5) | 0.309 | 2,933 (26.1) | 2,954 (26.3) | 0.004 |
| Calcium channel blockers | 998 (8.7) | 24,112 (8.7) | 0.002 | 984 (8.8) | 982 (8.8) | 0.001 |
| Antiarrhythmics | 5,305 (46.4) | 48,484 (17.4) | 0.653 | 5,085 (45.3) | 5,134 (45.8) | 0.009 |
| Lipid modifying agents | 2,122 (18.5) | 52,192 (18.7) | 0.005 | 2,092 (18.7) | 2,122 (18.9) | 0.007 |

**Abbreviations:** ACE, angiotensin converting enzyme; ALT, alanine aminotransferase; AST, aspartate aminotransferase; BMI, body mass index; CKD, chronic kidney disease; HbA_1C_, hemoglobin A**_1C_;** HDL, high-density lipoprotein; IHD, ischemic heart disease; n, number; LDL, low density lipoprotein, MBS, metabolic bariatric surgery; PSM, propensity score matching; SD, standard deviation; SLD, steatotic liver disease; SMD, standardized mean difference; T2D, type 2 diabetes.

† Includes: metformin, glipizide, sitagliptin, glimepiride, pioglitazone, glyburide, empagliflozin, dapagliflozin, acarbose, miglitol, canagliflozin, linagliptin, saxagliptin, alogliptin, ertugliflozin, chlorpropamide, nateglinide, repaglinide, rosiglitazone.

‡ Includes: liraglutide, semaglutide, dulaglutide, tirzepatide, exenatide.

**Table S18** Baseline characteristics of male patients with SLD with and without history of metabolic bariatric surgery.

| **Characteristic** | **Before PSM** | | | **After PSM** | | |
| --- | --- | --- | --- | --- | --- | --- |
|  | **MBS**  **n=3,373** | **No-MBS**  **n= 238,986** | **SMD** | **MBS n=3,334** | **No-MBS n=3,334** | **SMD** |
| Age, mean (SD), years | 48 [12] | 51 [15] | 0.254 | 48 [12] | 48 [14] | 0.029 |
| **Race & Ethnicity, No. (%)** | | | | | | |
| Asian | 39 (1.2) | 15,351 (6.5) | 0.280 | 39 (1.2) | 40 (1.2) | 0.003 |
| Black or African American | 280 (8.3) | 16,101 (6.8) | 0.058 | 277 (8.3) | 287 (8.6) | 0.011 |
| White | 2,626 (78.0) | 171,191 (72.1) | 0.137 | 2,599 (78.0) | 2,604 (78.1) | 0.004 |
| Hispanic or Latino | 457 (13.6) | 27,932 (11.8) | 0.055 | 454 (13.6) | 419 (12.6) | 0.031 |
| **Anthropometric and biochemical measurements, mean (SD)** | | | | | | |
| BMI, kg/m^2^ | 43.4 [8.9] | 32.6 [6.8] | 1.358 | 43.3 [8.8] | 42.4 [8.8] | 0.094 |
| HbA_1C_, *%* | 6.4 [1.4] | 6.5 [1.7] | 0.122 | 6.4 [1.4] | 6.6 [1.7] | 0.131 |
| ALT, U/L | 46 [44] | 62 [80] | 0.243 | 46 [44] | 50 [62] | 0.075 |
| AST, U/L | 33 [28] | 46 [97] | 0.181 | 33 [28] | 38 [57] | 0.099 |
| Triglycerides, mg/dL | 173 [143] | 199 [253] | 0.125 | 174 [143] | 179 [152] | 0.036 |
| HDL, mg/dL | 39 [12] | 41 [15] | 0.104 | 39 [12] | 38 [13] | 0.119 |
| LDL, mg/dL | 99 [35] | 105 [39] | 0.150 | 99 [35] | 99 [35] | 0.012 |
| **Comorbidities, No. (%)** | | | | | | |
| T2D | 1,327 (39.4) | 43,465 (18.3) | 0.479 | 1,307 (39.2) | 1,333 (40.0) | 0.016 |
| Hypertension | 2,389 (71.0) | 97,860 (41.2) | 0.387 | 2,359 (70.8) | 2,448 (73.4) | 0.060 |
| IHD | 429 (12.7) | 25,875 (10.9) | 0.057 | 428 (12.8) | 455 (13.6) | 0.024 |
| Cerebrovascular disease | 73 (2.2) | 7,251 (3.1) | 0.055 | 73 (2.2) | 86 (2.6) | 0.026 |
| Dyslipidemia | 1,865 (55.4) | 88,136 (37.1) | 0.232 | 1,838 (55.1) | 1,872 (56.1) | 0.021 |
| Smoking | 244 (7.2) | 22,677 (9.5) | 0.083 | 242 (7.3) | 256 (7.7) | 0.016 |
| CKD | 194 (5.8) | 10,209 (4.3) | 0.067 | 193 (5.8) | 199 (6.0) | 0.008 |

**Table S18 (continued)** Baseline characteristics of male patients with SLD with and without history of metabolic bariatric surgery.

| **Characteristic** | **Before PSM** | | | **After PSM** | | |
| --- | --- | --- | --- | --- | --- | --- |
|  | **MBS**  **n=3,373** | **No-MBS**  **n= 238,986** | **SMD** | **MBS n=3,334** | **No-MBS n=3,334** | **SMD** |
| **Medication, No. (%)** | | | | | | |
| Insulin | 810 (24.1) | 16,310 (6.9) | 0.490 | 792 (23.8) | 812 (24.4) | 0.014 |
| Oral Hypoglycemic^†^ | 681 (20.2) | 25,482 (10.7) | 0.265 | 673 (20.2) | 685 (20.5) | 0.009 |
| Other Hypoglycemic^‡^ | 132 (3.9) | 1,983 (0.8) | 0.204 | 126 (3.8) | 131 (3.9) | 0.008 |
| ACE inhibitors | 757 (22.5) | 31,714 (13.3) | 0.240 | 748 (22.4) | 794 (23.8) | 0.033 |
| Angiotensin II inhibitors | 380 (11.3) | 17,970 (7.6) | 0.128 | 374 (11.2) | 367 (11.0) | 0.007 |
| Beta-blockers | 1,155 (34.3) | 35,422 (14.9) | 0.462 | 1,134 (34.0) | 1,134 (34.0) | <0.001 |
| Calcium channel blockers | 479 (14.2) | 23,362 (9.8) | 0.135 | 471 (14.1) | 479 (14.4) | 0.007 |
| Antiarrhythmics | 1,560 (46.3) | 36,741 (15.5) | 0.709 | 1,527 (45.8) | 1,511 (45.3) | 0.010 |
| Lipid modifying agents | 885 (26.3) | 50,154 (21.1) | 0.122 | 877 (26.3) | 898 (26.9) | 0.014 |

**Abbreviations:** ACE, angiotensin converting enzyme; ALT, alanine aminotransferase; AST, aspartate aminotransferase; BMI, body mass index; CKD, chronic kidney disease; HbA_1C_, hemoglobin A**_1C_;** HDL, high-density lipoprotein; IHD, ischemic heart disease; n, number; LDL, low density lipoprotein, MBS, metabolic bariatric surgery; PSM, propensity score matching; SD, standard deviation; SLD, steatotic liver disease; SMD, standardized mean difference; T2D, type 2 diabetes.

† Includes: metformin, glipizide, sitagliptin, glimepiride, pioglitazone, glyburide, empagliflozin, dapagliflozin, acarbose, miglitol, canagliflozin, linagliptin, saxagliptin, alogliptin, ertugliflozin, chlorpropamide, nateglinide, repaglinide, rosiglitazone.

‡ Includes: liraglutide, semaglutide, dulaglutide, tirzepatide, exenatide.

**Table S19** Associations of sleeve gastrectomy with major hepatic and extra hepatic clinical outcomes in patients with SLD.

| **Outcome** | **Number of patients in MBS** | **Patients with outcome in MBS, No. (%)** | **Number of patients in no-MBS** | **Patients with outcome in no-MBS, No. (%)** | **Hazard Ratio (CI)** |
| --- | --- | --- | --- | --- | --- |
| **Hepatic outcomes** | | | | | |
| MALO | 5,190 | 132 (2.5) | 5,200 | 196 (3.8) | 0.67  (0.53-0.83) |
| Cirrhosis | 5,195 | 63 (1.2) | 5,200 | 81 (1.6) | 0.77  (0.55-1.07) |
| Decompensated cirrhosis | 5,195 | 74 (1.4) | 5,200 | 125 (2.4) | 0.59  (0.44-0.78) |
| HCC | 5,200 | ≤10^†^ (0.2) | 5,200 | ≤10^†^ (0.2) | 1.01  (0.29-3.47) |
| Intrahepatic cancer^‡^ | 5,199 | ≤10^†^ (0.2) | 5,194 | ≤10^†^ (0.2) | 0.50  (0.17-1.47) |
| Liver transplant | 5,200 | ≤10^†^ (0.2) | 5,200 | ≤10^†^ (0.2) | 0.20  (0.02-1.71) |
| **Cardiovascular outcomes** | | | | | |
| MACE | 4,842 | 169 (3.5) | 4,620 | 385 (8.3) | 0.41  (0.34-0.49) |
| Acute MI | 5,148 | 39 (0.8) | 5,084 | 111 (2.2) | 0.34  (0.24-0.50) |
| Stroke | 5,114 | 91 (1.8) | 5,067 | 179 (3.5) | 0.50  (0.39-0.64) |
| Heart failure | 4,946 | 93 (1.9) | 4,770 | 277 (5.8) | 0.32  (0.25-0.40) |
| **Renal outcomes** | | | | | |
| MAKE | 5,173 | 16 (0.3) | 5,146 | 54 (1.0) | 0.29  (0.17-0.51) |
| CKD5 | 5,190 | ≤10^†^ (0.2) | 5,185 | 17 (0.3) | 0.29  (0.11-0.79) |
| ESRF | 5,174 | 15 (0.3) | 5,161 | 34 (0.7) | 0.44  (0.24-0.80) |
| Dialysis | 5,186 | 14 (0.3) | 5,162 | 43 (0.8) | 0.32  (0.18-0.58) |
| **Obesity-related cancers** | | | | | |
| Composite | 5,054 | 99 (2.0) | 4,795 | 147 (3.1) | 0.63  (0.49-0.82) |
| Colorectal | 5,182 | ≤10^†^ (0.2) | 5,144 | 26 (0.5) | 0.34  (0.16-0.73) |
| Breast | 5,155 | 39 (0.8) | 5,036 | 58 (1.2) | 0.65  (0.43-0.98) |
| Uterine | 5,175 | 18 (0.3) | 5,115 | 24 (0.5) | 0.73  (0.40-1.35) |
| Ovarian | 5,190 | ≤10 ^a^ (0.2) | 5,183 | ≤10 ^a^ (0.2) | 0.75  (0.26-2.15) |
| Renal | 5,188 | ≤10 ^a^ (0.2) | 5,173 | 17 (0.3) | 0.29  (0.11-0.79) |

**Table S19 (continued)** Associations of sleeve gastrectomy with major hepatic and extra hepatic clinical outcomes in patients with SLD.

| **Outcome** | **Number of patients in MBS** | **Patients with outcome in MBS, No. (%)** | **Number of patients in no-MBS** | **Patients with outcome in no-MBS, No. (%)** | **Hazard Ratio (CI)** |
| --- | --- | --- | --- | --- | --- |
| **Obesity-related cancers (continued)** | | | | | |
| Pancreatic | 5,190 | ≤10^†^ (0.2) | 5,183 | ≤10^†^ (0.2) | 0.33  (0.09-1.22) |
| Gastric | 5,188 | ≤10^†^ (0.2) | 5,173 | 17 (0.3) | 0.98  (0.20-4.85) |
| Oesophagus | 5,197 | ≤10^†^ (0.2) | 5,180 | ≤10^†^ (0.2) | 0.79  (0.21-2.94) |
| Gallbladder | 5,191 | ≤10^†^ (0.2) | 5,198 | ≤10^†^ (0.2) | n/a |
| Thyroid | 5,200 | ≤10^†^ (0.2) | 5,195 | ≤10^†^ (0.2) | 1.26  (0.64-2.48) |
| Meningeal | 5,200 | 0 (0.0) | 5,200 | ≤10^†^ (0.2) | 0.33  (0.03-3.14) |
| Multiple myeloma | 5,166 | 19 (0.4) | 5,154 | 15 (0.3) | 0.50  (0.15-1.65) |
| **All-cause mortality** | 5,199 | 58 (1.1) | 5,199 | 217 (4.2) | 0.27  (0.20-0.35) |

**Abbreviations:** CI, confidence interval; CKD5, chronic kidney disease stage 5; ESRF, end stage renal failure; HCC, hepatocellular carcinoma; MALO, major adverse liver outcomes; MACE, major adverse cardiovascular outcomes; MAKE, major adverse kidney events; MALO, major adverse liver outcomes; MBS, metabolic bariatric surgery; MI, myocardial infarction; SLD, steatotic liver disease.

† To protect patients’ anonymity on TriNetX, if sample size is ≤10, the exact number of patients is concealed.

‡Intrahepatic cancer includes hepatocellular carcinoma and intrahepatic biliary tree cancers; intrahepatic cancer is a composite of MALO.

**Table S20** Associations of Roux-en-Y gastric bypass with major hepatic and extra hepatic clinical outcomes in patients with SLD.

| **Outcome** | **Number of patients in MBS** | **Patients with outcome in MBS, No. (%)** | **Number of patients in no-MBS** | **Patients with outcome in no-MBS, No. (%)** | **Hazard Ratio (CI)** |
| --- | --- | --- | --- | --- | --- |
| **Hepatic outcomes** | | | | | |
| MALO | 4,353 | 133 (3.1) | 4,368 | 175 (4.0) | 0.74  (0.59-0.93) |
| Cirrhosis | 4,361 | 40 (0.9) | 4,368 | 86 (2.0) | 0.45  (0.31-0.66) |
| Decompensated cirrhosis | 4,360 | 94 (2.2) | 4,368 | 113 (2.6) | 0.81  (0.62-1.08) |
| HCC | 4,368 | ≤10^†^ (0.2) | 4,368 | 13 (0.3) | 0.49  (0.09-2.66) |
| Intrahepatic cancer^‡^ | 4,368 | ≤10^†^ (0.2) | 4,364 | ≤10^†^ (0.2) | 0.49  (0.12-1.94) |
| Liver transplant | 4,368 | ≤10^†^ (0.2) | 4,368 | 0 (0.0) | n/a |
| **Cardiovascular outcomes** | | | | | |
| MACE | 4,071 | 151 (3.7) | 3,922 | 328 (8.4) | 0.42  (0.35-0.51) |
| Acute MI | 4,324 | 38 (0.9) | 4,280 | 86 (2.0) | 0.43  (0.29-0.62) |
| Stroke | 4,294 | 78 (1.8) | 4,257 | 144 (3.4) | 0.52  (0.39-0.68) |
| Heart failure | 4,160 | 79 (1.9) | 4,040 | 236 (5.8) | 0.31  (0.24-0.40) |
| **Renal outcomes** | | | | | |
| MAKE | 4,356 | 16 (0.4) | 4,338 | 44 (1.0) | 0.35  (0.20-0.63) |
| CKD5 | 4,365 | ≤10^†^ (0.2) | 4,364 | 12 (0.3) | 0.41  (0.14-1.15) |
| ESRF | 4,359 | ≤10^†^ (0.2) | 4,351 | 34 (0.8) | 0.29  (0.14-0.58) |
| Dialysis | 4,360 | 12 (0.3) | 4,345 | 37 (0.9) | 0.32  (0.16-0.60) |
| **Obesity-related cancers** | | | | | |
| Composite | 4,244 | 78 (1.8) | 3,993 | 137 (3.4) | 0.52  (0.39-0.68) |
| Colorectal | 4,357 | ≤10^†^ (0.2) | 4,327 | 21 (0.5) | 0.28  (0.11-0.68) |
| Breast | 4,315 | 24 (0.6) | 4,213 | 47 (1.1) | 0.48  (0.30-0.79) |
| Uterine | 4,350 | ≤10^†^ (0.2) | 4,300 | 26 (0.6) | 0.37  (0.18-0.77) |
| Ovarian | 4,358 | ≤10^†^ (0.2) | 4,340 | 11 (0.3) | 0.71  (0.28-1.76) |
| Renal | 4,357 | 12 (0.3) | 4,334 | 20 (0.5) | 0.58  (0.28-1.19) |

**Table S20 (continued)** Associations of Roux-en-Y gastric bypass with major hepatic and extra hepatic clinical outcomes in patients with SLD.

| **Outcome** | **Number of patients in MBS** | **Patients with outcome in MBS, No. (%)** | **Number of patients in no-MBS** | **Patients with outcome in no-MBS, No. (%)** | **Hazard Ratio (CI)** |
| --- | --- | --- | --- | --- | --- |
| **Obesity-related cancers (continued)** | | | | | |
| Pancreatic | 4,361 | ≤10^†^ (0.2) | 4,353 | ≤10^†^ (0.2) | 0.68  (0.26-1.78) |
| Gastric | 4,360 | ≤10^†^ (0.2) | 4,362 | ≤10^†^ (0.2) | 0.78  (0.21-2.90) |
| Oesophagus | 4,368 | ≤10^†^ (0.2) | 4,359 | ≤10^†^ (0.2) | 0.65  (0.11-3.86) |
| Gallbladder | 4,368 | 0 (0.0) | 4,366 | ≤10^†^ (0.2) | n/a |
| Thyroid | 4,356 | ≤10^†^ (0.2) | 4,331 | 15 (0.3) | 0.58  (0.25-1.33) |
| Meningeal | 4,368 | 0 (0.0) | 4,365 | ≤10^†^ (0.2) | n/a |
| Multiple myeloma | 4,367 | ≤10^†^ (0.2) | 4,357 | ≤10^†^ (0.2) | 0.14  (0.02-1.13) |
| **All-cause mortality** | 4,367 | 72 (1.6) | 4,368 | 199 (4.6) | 0.35  (0.27-0.46) |

**Abbreviations:** CI, confidence interval; CKD5, chronic kidney disease stage 5; ESRF, end stage renal failure; HCC, hepatocellular carcinoma; MALO, major adverse liver outcomes; MACE, major adverse cardiovascular outcomes; MAKE, major adverse kidney events; MALO, major adverse liver outcomes; MBS, metabolic bariatric surgery; MI, myocardial infarction; SLD, steatotic liver disease.

† To protect patients’ anonymity on TriNetX, if sample size is ≤10, the exact number of patients is concealed.

‡Intrahepatic cancer includes hepatocellular carcinoma and intrahepatic biliary tree cancers; intrahepatic cancer is a composite of MALO.

**
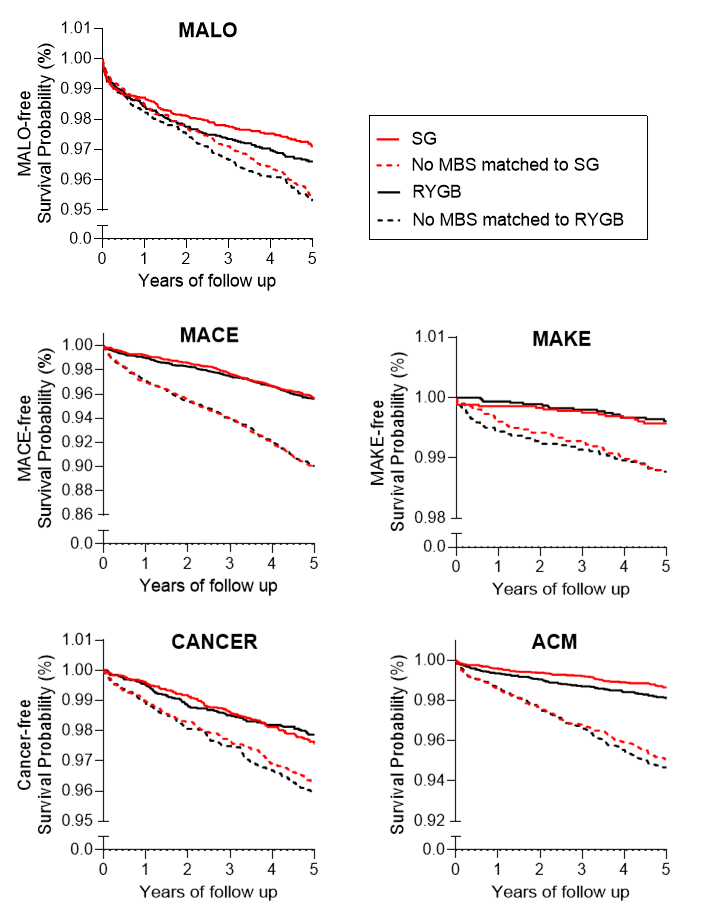
Figure S2** Event-free probability for MBS and no-MBS cohorts depending on MBS type. **Abbreviations**: ACM, all-cause mortality; CANCER, obesity-associated cancers; MACE, major adverse cardiovascular events; MAKE, major adverse kidney events; MALO, major adverse liver outcomes; RYGB, Roux-en-Y gastric bypass; SG, sleeve gastrectomy.

**Table S21** Associations of MBS with major hepatic and extra hepatic clinical outcomes in patients with SLD and T2D.

| **Outcome** | **Number of patients in MBS** | **Patients with outcome in MBS, No. (%)** | **Number of patients in no-MBS** | **Patients with outcome in no-MBS, No. (%)** | **Hazard Ratio (CI)** |
| --- | --- | --- | --- | --- | --- |
| **Hepatic outcomes** | | | | | |
| MALO | 5,757 | 246 (4.3) | 5,491 | 308 (5.6) | 0.75  (0.64-0.89) |
| Cirrhosis | 5,757 | 136 (2.4) | 5,589 | 180 (3.2) | 0.72  (0.58-0.90) |
| Decompensated cirrhosis | 5,757 | 129 (2.2) | 5,619 | 197 (3.5) | 0.63  (0.51-0.79) |
| HCC | 5,757 | ≤10^†^ (0.2) | 5,748 | 22 (0.4) | 0.09  (0.02-0.38) |
| Intrahepatic cancer^‡^ | 5,754 | ≤10^†^ (0.2) | 5,740 | 31 (0.5) | 0.19  (0.08-0.46) |
| Liver transplant | 5,757 | ≤10^†^ (0.2) | 5,753 | ≤10^†^ (0.2) | 0.70  (0.22-2.21) |
| **Cardiovascular outcomes** | | | | | |
| MACE | 5,092 | 325 (6.4) | 4,841 | 579 (12.0) | 0.52  (0.45-0.59) |
| Acute MI | 5,636 | 97 (1.7) | 5,563 | 169 (3.0) | 0.56  (0.43-0.71) |
| Stroke | 5,575 | 189 (3.4) | 5,514 | 299 (5.4) | 0.61  (0.51-0.73) |
| Heart failure | 5,306 | 202 (3.8) | 5,105 | 428 (8.4) | 0.44  (0.38-0.52) |
| **Renal outcomes** | | | | | |
| MAKE | 5,710 | 57 (1.0) | 5,661 | 92 (1.6) | 0.61  (0.44-0.84) |
| CKD5 | 5,743 | 25 (0.4) | 5,736 | 31 (0.5) | 0.80  (0.47-1.35) |
| ESRF | 5,715 | 40 (0.7) | 5,686 | 68 (1.2) | 0.58  (0.39-0.85) |
| Dialysis | 5,724 | 40 (0.7) | 5,690 | 63 (1.1) | 0.62  (0.42-0.93) |
| **Obesity-related cancers** | | | | | |
| Composite | 5,521 | 132 (2.4) | 5,238 | 194 (3.7) | 0.63  (0.51-0.79) |
| Colorectal | 5,732 | 17 (0.3) | 5,687 | 27 (0.5) | 0.62  (0.34-1.13) |
| Breast | 5,672 | 39 (0.7) | 5,598 | 52 (0.9) | 0.73  (0.48-1.11) |
| Uterine | 5,722 | 20 (0.3) | 5,655 | 40 (0.7) | 0.49  (0.28-0.83) |
| Ovarian | 5,737 | 14 (0.2) | 5,724 | ≤10^†^ (0.2) | 1.73  (0.72-4.12) |
| Renal | 5,733 | 22 (0.4) | 5,700 | 38 (0.7) | 0.57  (0.34-0.96) |

**Table S21 (continued)**Associations of MBS with major hepatic and extra hepatic clinical outcomes in patients with SLD and T2D.

| **Outcome** | **Number of patients in MBS** | **Patients with outcome in MBS, No. (%)** | **Number of patients in no-MBS** | **Patients with outcome in no-MBS, No. (%)** | **Hazard Ratio (CI)** |
| --- | --- | --- | --- | --- | --- |
| **Obesity-related cancers (continued)** | | | | | |
| Pancreatic | 5,749 | 14 (0.2) | 5,731 | 20 (0.3) | 0.69  (0.35-1.36) |
| Gastric | 5,743 | ≤10^†^ (0.2) | 5,752 | ≤10^†^ (0.2) | 0.61  (0.20-1.88) |
| Oesophagus | 5,755 | ≤10^†^ (0.2) | 5,748 | ≤10^†^ (0.2) | 4.94  (0.58-42.30) |
| Gallbladder | 5,757 | 0 (0.0) | 5,756 | ≤10^†^ (0.2) | n/a |
| Thyroid | 5,719 | 16 (0.3) | 5,694 | 12 (0.2) | 1.31  (0.62-2.77) |
| Meningeal | 5,756 | 0 (0.0) | 5,757 | 0 (0.0) | n/a |
| Multiple myeloma | 5,752 | ≤10^†^ (0.2) | 5,738 | 11 (0.2) | 0.45  (0.16-1.29) |
| **All-cause mortality** | 5,757 | 171 (3.0) | 5,703 | 380 (6.7) | 0.44  (0.37-0.53) |

**Abbreviations:** CI, confidence interval; CKD5, chronic kidney disease stage 5; ESRF, end stage renal failure; HCC, hepatocellular carcinoma; MALO, major adverse liver outcomes; MACE, major adverse cardiovascular outcomes; MAKE, major adverse kidney events; MALO, major adverse liver outcomes; MBS, metabolic bariatric surgery; MI, myocardial infarction; SLD, steatotic liver disease.

† To protect patients’ anonymity on TriNetX, if sample size is ≤10, the exact number of patients is concealed.

‡Intrahepatic cancer includes hepatocellular carcinoma and intrahepatic biliary tree cancers; intrahepatic cancer is a composite of MALO.

**Table S22** Associations of MBS with major hepatic and extra hepatic clinical outcomes in patients with SLD and without T2D.

| **Outcome** | **Number of patients in MBS** | **Patients with outcome in MBS, No. (%)** | **Number of patients in no-MBS** | **Patients with outcome in no-MBS, No. (%)** | **Hazard Ratio (CI)** |
| --- | --- | --- | --- | --- | --- |
| **Hepatic outcomes** | | | | | |
| MALO | 5,968 | 140 (2.3) | 5,967 | 136 (2.3) | 1.01  (0.80-1.28) |
| Cirrhosis | 5,968 | 39 (0.7) | 5,967 | 49 (0.8) | 0.78  (0.51-1.19) |
| Decompensated cirrhosis | 5,968 | 107 (1.8) | 5,968 | 96 (1.6) | 1.09  (0.83-1.44) |
| HCC | 5,968 | ≤10^†^ (0.2) | 5,968 | ≤10^†^ (0.2) | 0.09  (0.307-28.37) |
| Intrahepatic cancer^‡^ | 5,962 | ≤10^†^ (0.2) | 5,962 | ≤10^†^ (0.2) | 0.59  (0.21-1.62) |
| Liver transplant | 5,968 | ≤10^†^ (0.2) | 5,968 | ≤10^†^ (0.2) | 0.49  (0.05-5.45) |
| **Cardiovascular outcomes** | | | | | |
| MACE | 5,784 | 84 (1.5) | 5,777 | 147 (2.5) | 0.56  (0.43-0.73) |
| Acute MI | 5,916 | 41 (0.7) | 5,910 | 57 (1.0) | 0.71  (0.47-1.06) |
| Stroke | 5,883 | 66 (1.1) | 5,869 | 121 (2.1) | 0.53  (0.39-0.72) |
| Heart failure | 5,784 | 84 (1.5) | 5,777 | 147 (2.5) | 0.56  (0.43-0.73) |
| **Renal outcomes** | | | | | |
| MAKE | 5,958 | 10 (0.2) | 5,952 | 15 (0.3) | 0.26  (0.09-0.79) |
| CKD5 | 5,964 | ≤10^†^  (0.2) | 5,966 | ≤10^†^  (0.2) | 0.16  (0.02-1.36) |
| ESRF | 5,961 | ≤10^†^  (0.2) | 5,956 | ≤10^†^  (0.2) | 0.25  (0.05-1.16) |
| Dialysis | 5,962 | ≤10^†^  (0.2) | 5,957 | 12 (0.2) | 0.49  (0.19-1.31) |
| **Obesity-related cancers** | | | | | |
| Composite | 5,770 | 81 (1.4) | 5,589 | 134 (2.4) | 0.57  (0.43-0.75) |
| Colorectal | 5,941 | ≤10^†^ (0.2) | 5,907 | 22 (0.4) | 0.40  (0.18-0.87) |
| Breast | 5,903 | 35 (0.6) | 5,815 | 40 (0.7) | 0.84  (0.54-1.33) |
| Uterine | 5,947 | ≤10^†^ (0.2) | 5,909 | 15 (0.3) | 0.52  (0.22-1.23) |
| Ovarian | 5,957 | ≤10^†^ (0.2) | 5,939 | 13 (0.2) | 0.45  (0.17-1.19) |
| Renal | 5,941 | ≤10^†^  (0.2) | 5,938 | 20 (0.3) | 0.24  (0.09-0.65) |

**Table S22 (continued)** Associations of MBS with major hepatic and extra hepatic clinical outcomes in patients with SLD and without T2D.

| **Outcome** | **Number of patients in MBS** | **Patients with outcome in MBS, No. (%)** | **Number of patients in no-MBS** | **Patients with outcome in no-MBS, No. (%)** | **Hazard Ratio (CI)** |
| --- | --- | --- | --- | --- | --- |
| **Obesity-related cancers (continued)** | | | | | |
| Pancreatic | 5,958 | ≤10^†^ (0.2) | 5,958 | 13 (0.2) | 0.15  (0.03-0.68) |
| Gastric | 5,948 | ≤10^†^ (0.2) | 5,960 | ≤10^†^ (0.2) | 1.96  (0.49-7.85) |
| Oesophagus | 5,966 | ≤10^†^ (0.2) | 5,963 | ≤10^†^ (0.2) | 0.74  (0.17-3.31) |
| Gallbladder | 5,968 | ≤10^†^  (0.2) | 5,964 | ≤10^†^ (0.2) | 0.98  (0.06-15.71) |
| Thyroid | 5,946 | 11 (0.2) | 5,927 | 17 (0.3) | 0.63  (0.30-1.35) |
| Meningeal | 5,968 | 0 (0.0) | 5,968 | ≤10^†^  (0.2) | n/a |
| Multiple myeloma | 5,964 | ≤10^†^ (0.2) | 5,962 | ≤10^†^  (0.2) | 0.61  (0.20-1.87) |
| **All-cause mortality** | 5,968 | 114 (1.9) | 5,967 | 208 (3.5) | 0.54  (0.43-0.68) |

**Abbreviations:** CI, confidence interval; CKD5, chronic kidney disease stage 5; ESRF, end stage renal failure; GLP1- glucagon like peptide 1; HCC, hepatocellular carcinoma; MALO, major adverse liver outcomes; MACE, major adverse cardiovascular outcomes; MAKE, major adverse kidney events; MALO, major adverse liver outcomes; MBS, metabolic bariatric surgery; MI, myocardial infarction; SLD, steatotic liver disease.

† To protect patients’ anonymity on TriNetX, if sample size is ≤10, the exact number of patients is concealed.

‡Intrahepatic cancer includes hepatocellular carcinoma and intrahepatic biliary tree cancers; intrahepatic cancer is a composite of MALO.

**
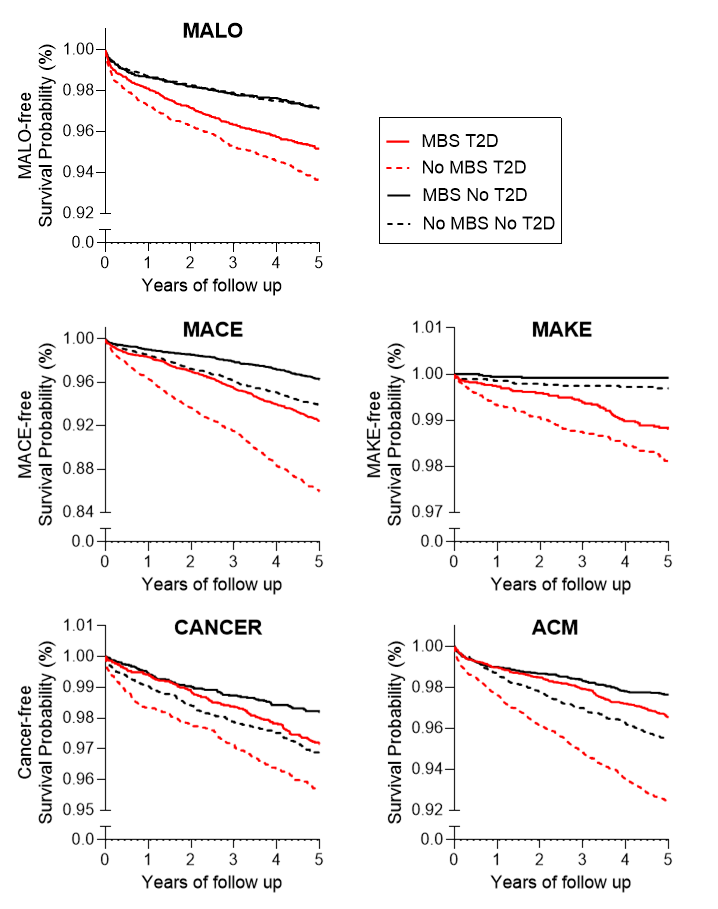
 Figure S3** Event-free probability for MBS and no-MBS cohorts with or without T2D. **Abbreviations**: ACM, all-cause mortality; CANCER, obesity-associated cancers; MACE, major adverse cardiovascular events; MAKE, major adverse kidney events; MALO, major adverse liver outcomes; T2D, type 2 diabetes.

**Table S23** Associations of MBS with major hepatic and extra hepatic clinical outcomes in patients with SLD and BMI≥50kg/m^2^.

| **Outcome** | **Number of patients in MBS** | **Patients with outcome in MBS, No. (%)** | **Number of patients in no-MBS** | **Patients with outcome in no-MBS, No. (%)** | **Hazard Ratio (CI)** |
| --- | --- | --- | --- | --- | --- |
| **Hepatic outcomes** | | | | | |
| MALO | 2,148 | 65 (3.0) | 2,025 | 87 (4.3) | 0.69  (0.50-0.96) |
| Cirrhosis | 2,148 | 28 (1.3) | 2,074 | 61 (2.9) | 0.43  (0.28-0.68) |
| Decompensated cirrhosis | 2,148 | 40 (1.9) | 2,069 | 51 (2.5) | 0.75  (0.50-1.13) |
| HCC | 2,148 | 0 (0.0) | 2,143 | ≤10^†^ (0.5) | n/a |
| Intrahepatic cancer^‡^ | 2,148 | ≤10^†^ (0.5) | 2,141 | ≤10^†^ (0.5) | 0.28  (0.06-1.34) |
| Liver transplant | 2,148 | 0 (0.0) | 2,146 | ≤10^†^ (0.5) | n/a |
| **Cardiovascular outcomes** | | | | | |
| MACE | 1,935 | 96 (5.0) | 1,831 | 193 (10.5) | 0.46  (0.36-0.59) |
| Acute MI | 2,121 | 23 (1.1) | 2,101 | 44 (2.1) | 0.51  (0.31-0.84) |
| Stroke | 2,109 | 37 (1.8) | 2,087 | 78 (3.7) | 0.46  (0.31-0.68) |
| Heart failure | 1,976 | 65 (3.3) | 1,888 | 149 (7.9) | 0.41  (0.31-0.55) |
| **Renal outcomes** | | | | | |
| MAKE | 2,138 | 11 (0.5) | 2,117 | 45 (2.1) | 0.24  (0.12-0.46) |
| CKD5 | 2,145 | ≤10^†^ (0.5) | 2,146 | 14 (0.7) | 0.07  (0.01-0.53) |
| ESRF | 2,139 | ≤10^†^ (0.5) | 2,125 | 26 (1.2) | 0.11  (0.03-0.37) |
| Dialysis | 2,143 | 11 (0.5) | 2,121 | 29 (1.4) | 0.37  (0.18-0.74) |
| **Obesity-related cancers** | | | | | |
| Composite | 2,089 | 35 (1.7) | 1,976 | 70 (3.5) | 0.46  (0.31-0.69) |
| Colorectal | 2,144 | ≤10^†^ (0.5) | 2,120 | ≤10^†^ (0.5) | 0.73  (0.25-2.09) |
| Breast | 2,129 | ≤10^†^ (0.5) | 2,100 | 20 (1.0) | 0.24  (0.09-0.64) |
| Uterine | 2,135 | ≤10^†^ (0.5) | 2,106 | 21 (1.0) | 0.46  (0.22-0.98) |
| Ovarian | 2,146 | ≤10^†^ (0.5) | 2,134 | ≤10^†^ (0.5) | 0.49  (0.12-1.96) |
| Renal | 2,141 | ≤10^†^ (0.5) | 2,126 | ≤10^†^ (0.5) | 0.32  (0.09-1.20) |

**Table S23 (continued)** Associations of MBS with major hepatic and extra hepatic clinical outcomes in patients with SLD and BMI≥50kg/m^2^.

| **Outcome** | **Number of patients in MBS** | **Patients with outcome in MBS, No. (%)** | **Number of patients in no-MBS** | **Patients with outcome in no-MBS, No. (%)** | **Hazard Ratio (CI)** |
| --- | --- | --- | --- | --- | --- |
| **Obesity-related cancers (continued)** | | | | | |
| Pancreatic | 2,145 | ≤10^†^ (0.5) | 2,145 | ≤10^†^ (0.5) | 0.98  (0.32-3.03) |
| Gastric | 2,146 | ≤10^†^ (0.5) | 2,147 | ≤10^†^ (0.5) | 0.98  (0.06-15.63) |
| Oesophagus | 2,147 | ≤10^†^ (0.5) | 2,148 | ≤10^†^ (0.5) | 0.49  (0.04-5.39) |
| Gallbladder | 2,148 | 0 (0.0) | 2,148 | ≤10^†^ (0.5) | n/a |
| Thyroid | 2,138 | ≤10^†^ (0.5) | 2,127 | ≤10^†^ (0.5) | 0.29  (0.08-1.06) |
| Meningeal | 2,148 | 0 (0.0) | 2,147 | ≤10^†^ (0.5) | n/a |
| Multiple myeloma | 2,148 | ≤10^†^ (0.5) | 2,143 | ≤10^†^ (0.5) | 1.96  (0.18-21.66) |
| **All-cause mortality** | 2,148 | 42 (2.0) | 2,097 | 142 (6.8) | 0.28  (0.20-0.40) |

**Abbreviations:** CI, confidence interval; CKD5, chronic kidney disease stage 5; ESRF, end stage renal failure; HCC, hepatocellular carcinoma; MALO, major adverse liver outcomes; MACE, major adverse cardiovascular outcomes; MAKE, major adverse kidney events; MALO, major adverse liver outcomes; MBS, metabolic bariatric surgery; MI, myocardial infarction; SLD, steatotic liver disease.

† To protect patients’ anonymity on TriNetX, if sample size is ≤10, the exact number of patients is concealed.

‡Intrahepatic cancer includes hepatocellular carcinoma and intrahepatic biliary tree cancers; intrahepatic cancer is a composite of MALO.

**Table S24** Associations of MBS with major hepatic and extra hepatic clinical outcomes in patients with SLD and BMI<50kg/m^2^.

| **Outcome** | **Number of patients in MBS** | **Patients with outcome in MBS, No. (%)** | **Number of patients in no-MBS** | **Patients with outcome in no-MBS, No. (%)** | **Hazard Ratio (CI)** |
| --- | --- | --- | --- | --- | --- |
| **Hepatic outcomes** | | | | | |
| MALO | 9,045 | 344 (3.8) | 8,582 | 365 (4.3) | 0.89  (0.77-1.02) |
| Cirrhosis | 9,045 | 138 (1.5) | 8,796 | 202 (2.3) | 0.66  (0.53-0.81) |
| Decompensated cirrhosis | 9,045 | 224 (2.5) | 8,766 | 252 (2.9) | 0.85  (0.71-1.02) |
| HCC | 9,045 | ≤10^†^  (0.1) | 9,030 | 19 (0.2) | 0.21  (0.07-0.61) |
| Intrahepatic cancer^‡^ | 9,036 | ≤10^†^ (0.1) | 9,018 | 38 (0.4) | 0.23  (0.11-0.48) |
| Liver transplant | 9,045 | ≤10^†^ (0.1) | 9,040 | 13 (0.1) | 0.15  (0.03-0.67) |
| **Cardiovascular outcomes** | | | | | |
| MACE | 8,277 | 421 (5.1) | 8,053 | 754 (9.4) | 0.53  (0.47-0.59) |
| Acute MI | 8,892 | 128 (1.4) | 8,846 | 206 (2.3) | 0.61  (0.49-0.76) |
| Stroke | 8,796 | 239 (2.7) | 8,771 | 336 (3.8) | 0.70  (0.59-0.82) |
| Heart failure | 8,568 | 231 (2.7) | 8,349 | 517 (6.2) | 0.42  (0.36-0.50) |
| **Renal outcomes** | | | | | |
| MAKE | 8,999 | 51 (0.6) | 8,959 | 99 (1.1) | 0.51  (0.36-0.71) |
| CKD5 | 9,035 | 20 (0.2) | 9,031 | 23 (0.3) | 0.86  (0.47-1.56) |
| ESRF | 9,005 | 34 (0.4) | 8,987 | 66 (0.7) | 0.51  (0.34-0.77) |
| Dialysis | 9,014 | 37 (0.4) | 8,978 | 73 (0.8) | 0.50  (0.34-0.74) |
| **Obesity-related cancers** | | | | | |
| Composite | 8,598 | 187 (2.2) | 8,173 | 277 (3.4) | 0.63  (0.52-0.76) |
| Colorectal | 8,991 | 23 (0.3) | 8,908 | 40 (0.4) | 0.56  (0.34-0.94) |
| Breast | 8,874 | 62 (0.7) | 8,691 | 91 (1.0) | 0.66  (0.48-0.91) |
| Uterine | 9,005 | 21 (0.2) | 8,911 | 53 (0.6) | 0.39  (0.23-0.64) |
| Ovarian | 9,020 | 14 (0.2) | 8,975 | 17 (0.2) | 0.81  (0.40-1.64) |
| Renal | 8,991 | 28 (0.3) | 8,965 | 45 (0.5) | 0.61  (0.38-0.98) |

**Table S24 (continued)** Associations of MBS with major hepatic and extra hepatic clinical outcomes in patients with SLD and BMI<50kg/m^2^.

| **Outcome** | **Number of patients in MBS** | **Patients with outcome in MBS, No. (%)** | **Number of patients in no-MBS** | **Patients with outcome in no-MBS, No. (%)** | **Hazard Ratio (CI)** |
| --- | --- | --- | --- | --- | --- |
| **Obesity-related cancers (continued)** | | | | | |
| Pancreatic | 9,023 | 13 (0.1) | 9,004 | 26 (0.3) | 0.49  (0.25-0.96) |
| Gastric | 9,007 | ≤10^†^ (0.1) | 9,032 | ≤10^†^ (0.1) | 1.10  (0.45-2.70) |
| Oesophagus | 9,040 | ≤10^†^ (0.1) | 9,031 | ≤10^†^ (0.1) | 0.99  (0.37-2.63) |
| Gallbladder | 9,045 | ≤10^†^  (0.1) | 9,042 | ≤10^†^ (0.1) | 0.98  (0.06-15.72) |
| Thyroid | 8,989 | 32 (0.4) | 8,955 | 28 (0.3) | 1.12  (0.68-1.86) |
| Meningeal | 9,044 | ≤10^†^  (0.1) | 9,044 | ≤10^†^ (0.1) | 0.14  (0.02-1.14) |
| Multiple myeloma | 9,034 | ≤10^†^ (0.1) | 9,018 | 14 (0.2) | 0.70  (0.31-1.58) |
| **All-cause mortality** | 9,045 | 237 (2.6) | 8,910 | 522 (5.9) | 0.44  (0.38-0.51) |

**Abbreviations:** CI, confidence interval; CKD5, chronic kidney disease stage 5; ESRF, end stage renal failure; HCC, hepatocellular carcinoma; MALO, major adverse liver outcomes; MACE, major adverse cardiovascular outcomes; MAKE, major adverse kidney events; MALO, major adverse liver outcomes; MBS, metabolic bariatric surgery; MI, myocardial infarction; SLD, steatotic liver disease.

† To protect patients’ anonymity on TriNetX, if sample size is ≤10, the exact number of patients is concealed.

‡Intrahepatic cancer includes hepatocellular carcinoma and intrahepatic biliary tree cancers; intrahepatic cancer is a composite of MALO.

**
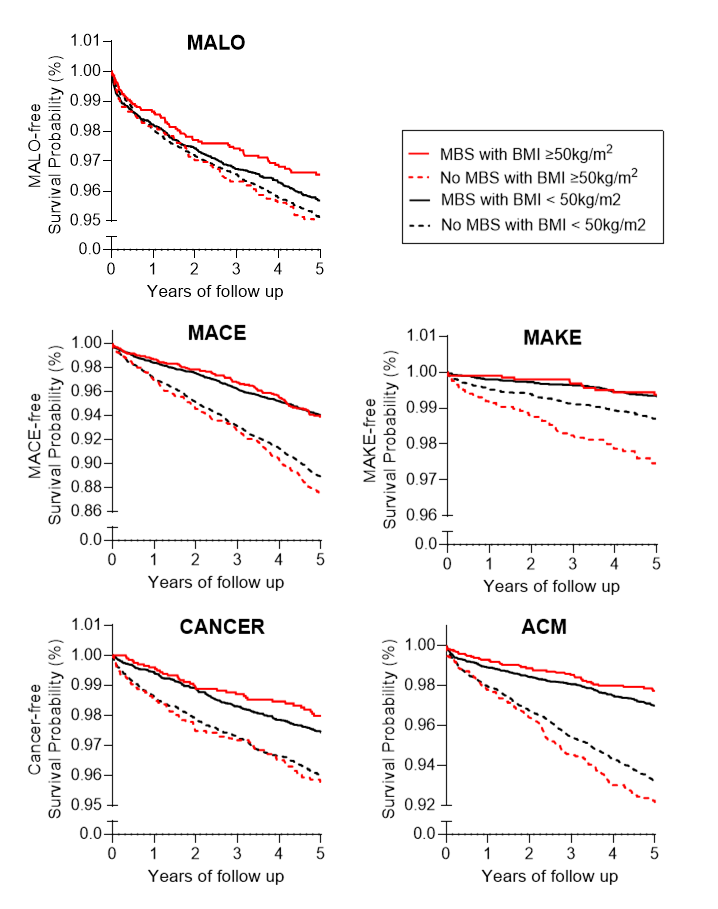
 Figure S4** Event-free probability for MBS and no-MBS cohorts depending on BMI. **Abbreviations**: ACM, all-cause mortality; BMI, body mass index; CANCER, obesity-associated cancers; MACE, major adverse cardiovascular events; MAKE, major adverse kidney events; MALO, major adverse liver outcomes.

**Table S25** Associations of MBS with major hepatic and extra hepatic clinical outcomes in female patients with SLD.

| **Outcome** | **Number of patients in MBS** | **Patients with outcome in MBS, No. (%)** | **Number of patients in no-MBS** | **Patients with outcome in no-MBS, No. (%)** | **Hazard Ratio (CI)** |
| --- | --- | --- | --- | --- | --- |
| **Hepatic outcomes** | | | | | |
| MALO | 11,217 | 371 (3.3) | 11,212 | 432 (3.9) | 0.85  (0.74-0.98) |
| Cirrhosis | 11,217 | 157 (1.4) | 11,214 | 194 (1.7) | 0.80  (0.65-0.99) |
| Decompensated cirrhosis | 11,217 | 235 (2.1) | 11,215 | 269 (2.4) | 0.87  (0.73-1.03) |
| HCC | 11,217 | ≤10^†^ (0.1) | 11,216 | 13 (0.1) | 0.38  (0.14-1.07) |
| Intrahepatic cancer^‡^ | 11,211 | ≤10^†^ (0.1) | 11,202 | 26 (0.2) | 0.27  (0.12-0.61) |
| Liver transplant | 11,217 | ≤10^†^ (0.1) | 11,217 | ≤10^†^ (0.1) | 0.33  (0.09-1.22) |
| **Cardiovascular outcomes** | | | | | |
| MACE | 10,472 | 483 (4.6) | 10,221 | 856 (8.4) | 0.54  (0.48-0.60) |
| Acute MI | 11,083 | 127 (1.1) | 11,040 | 197 (1.8) | 0.64  (0.51-0.80) |
| Stroke | 10,976 | 255 (2.3) | 10,923 | 416 (3.8) | 0.60  (0.51-0.70) |
| Heart failure | 10,755 | 281 (2.6) | 10,527 | 563 (5.3) | 0.48  (0.42-0.55) |
| **Renal outcomes** | | | | | |
| MAKE | 11,182 | 58 (0.5) | 11,141 | 94 (0.8) | 0.61  (0.44-0.85) |
| CKD5 | 11,205 | 19 (0.2) | 11,202 | 27 (0.2) | 0.70  (0.39-1.26) |
| ESRF | 11,186 | 39 (0.3) | 11,159 | 59 (0.5) | 0.66  (0.44-0.98) |
| Dialysis | 11,197 | 44 (0.4) | 11,163 | 65 (0.6) | 0.67  (0.46-0.98) |
| **Obesity-related cancers** | | | | | |
| Composite | 10,756 | 244 (2.3) | 10,352 | 399 (3.9) | 0.58  (0.49-0.68) |
| Colorectal | 11,179 | 24 (0.2) | 11,118 | 42 (0.4) | 0.56  (0.34-0.93) |
| Breast | 11,026 | 94 (0.9) | 10,849 | 155 (1.4) | 0.59  (0.46-0.76) |
| Uterine | 11,152 | 44 (0.4) | 11,065 | 78 (0.7) | 0.55  (0.38-0.80) |
| Ovarian | 11,187 | 22 (0.2) | 11,158 | 39 (0.3) | 0.56  (0.33-0.94) |
| Renal | 11,175 | 24 (0.2) | 11,152 | 43 (0.4) | 0.55  (0.33-0.91) |

**Table S25 (continued)** Associations of MBS with major hepatic and extra hepatic clinical outcomes in female patients with SLD.

| **Outcome** | **Number of patients in MBS** | **Patients with outcome in MBS, No. (%)** | **Number of patients in no-MBS** | **Patients with outcome in no-MBS, No. (%)** | **Hazard Ratio (CI)** |
| --- | --- | --- | --- | --- | --- |
| **Obesity-related cancers (continued)** | | | | | |
| Pancreatic | 11,199 | 15 (0.1) | 11,184 | 31 (0.3) | 0.48  (0.26-0.89) |
| Gastric | 11,194 | ≤10^†^ (0.1) | 11,210 | 12 (0.1) | 0.50  (0.19-1.32) |
| Oesophagus | 11,215 | ≤10^†^ (0.1) | 11,209 | ≤10^†^ (0.1) | 0.43  (0.11-1.64) |
| Gallbladder | 11,217 | 0 (0.0) | 11,215 | ≤10^†^ (0.1) | n/a |
| Thyroid | 11,144 | 38 (0.3) | 11,108 | 51 (0.5) | 0.74  (0.48-1.12) |
| Meningeal | 11,216 | ≤10^†^ (0.1) | 11,214 | ≤10^†^ (0.1) | 0.33  (0.03-3.16) |
| Multiple myeloma | 11,210 | ≤10^†^ (0.1) | 11,199 | 21 (0.2) | 0.42  (0.19-0.92) |
| **All-cause mortality** | 11,217 | 214 (1.9) | 11,216 | 451 (4.0) | 0.47  (0.40-0.55) |

**Abbreviations:** CI, confidence interval; CKD5, chronic kidney disease stage 5; ESRF, end stage renal failure; HCC, hepatocellular carcinoma; MALO, major adverse liver outcomes; MACE, major adverse cardiovascular outcomes; MAKE, major adverse kidney events; MALO, major adverse liver outcomes; MBS, metabolic bariatric surgery; MI, myocardial infarction; SLD, steatotic liver disease.

† To protect patients’ anonymity on TriNetX, if sample size is ≤10, the exact number of patients is concealed.

‡Intrahepatic cancer includes hepatocellular carcinoma and intrahepatic biliary tree cancers; intrahepatic cancer is a composite of MALO.

**Table S26** Associations of MBS with major hepatic and extra hepatic clinical outcomes in male patients with SLD.

| **Outcome** | **Number of patients in MBS** | **Patients with outcome in MBS, No. (%)** | **Number of patients in no-MBS** | **Patients with outcome in no-MBS, No. (%)** | **Hazard Ratio (CI)** |
| --- | --- | --- | --- | --- | --- |
| **Hepatic outcomes** | | | | | |
| MALO | 3,334 | 105 (3.1) | 3,333 | 132 (4.0) | 0.79  (0.61-1.02) |
| Cirrhosis | 3,334 | 46 (1.4) | 3,333 | 69 (2.1) | 0.66  (0.45-0.96) |
| Decompensated cirrhosis | 3,334 | 66 (2.0) | 3,334 | 83 (2.5) | 0.79  (0.57-1.09) |
| HCC | 3,334 | ≤10^†^ (0.3) | 3,334 | 13 (0.4) | 0.67  (0.11-4.01) |
| Intrahepatic cancer^‡^ | 3,331 | ≤10^†^ (0.3) | 3,331 | ≤10^†^ (0.3) | 0.30  (0.08-1.10) |
| Liver transplant | 3,334 | ≤10^†^ (0.3) | 3,334 | ≤10^†^ (0.3) | 1.01  (0.25-4.03) |
| **Cardiovascular outcomes** | | | | | |
| MACE | 2,957 | 147 (5.0) | 2,899 | 292 (10.1) | 0.48  (0.40-0.59) |
| Acute MI | 3,264 | 47 (1.4) | 3,225 | 93 (2.9) | 0.50  (0.35-0.71) |
| Stroke | 3,255 | 78 (2.4) | 3,224 | 137 (4.2) | 0.56  (0.43-0.74) |
| Heart failure | 3,057 | 90 (2.9) | 3,030 | 206 (6.8) | 0.43  (0.33-0.55) |
| **Renal outcomes** | | | | | |
| MAKE | 3,305 | 16 (0.5) | 3,291 | 36 (1.1) | 0.44  (0.24-0.79) |
| CKD5 | 3,327 | ≤10^†^ (0.3) | 3,323 | 15 (0.5) | 0.33  (0.12-0.91) |
| ESRF | 3,308 | 12 (0.4) | 3,300 | 27 (0.8) | 0.44  (0.22-0.87) |
| Dialysis | 3,313 | 11 (0.3) | 3,303 | 32 (1.0) | 0.34  (0.17-0.68) |
| **Obesity-related cancers** | | | | | |
| Composite | 3,268 | 36 (1.1) | 3,202 | 55 (1.7) | 0.64  (0.42-0.97) |
| Colorectal | 3,319 | ≤10^†^ (0.3) | 3,290 | 17 (0.5) | 0.52  (0.23-1.17) |
| Breast | 3,334 | 0 (0) | 3,333 | ≤10^†^ (0.3) | n/a |
| Renal | 3,314 | ≤10^†^ (0.3) | 3,291 | 17 (0.5) | 0.52  (0.23-1.17) |
| Pancreatic | 3,327 | ≤10^†^ (0.3) | 3,319 | ≤10^†^ (0.3) | 1.00  (0.32-3.10) |
| Gastric | 3,317 | ≤10^†^ (0.3) | 3,327 | ≤10^†^ (0.3) | 0.42  (0.11-1.63) |

**Table S26 (continued)** Associations of MBS with major hepatic and extra hepatic clinical outcomes in male patients with SLD.

| **Outcome** | **Number of patients in MBS** | **Patients with outcome in MBS, No. (%)** | **Number of patients in no-MBS** | **Patients with outcome in no-MBS, No. (%)** | **Hazard Ratio (CI)** |
| --- | --- | --- | --- | --- | --- |
| **Obesity-related cancers (continued)** | | | | | |
| Oesophagus | 3,331 | ≤10^†^ (0.3) | 3,321 | ≤10^†^ (0.3) | 2.30  (0.60-8.91) |
| Gallbladder | 3,334 | 0 (0) | 3,334 | 0 (0) | n/a |
| Thyroid | 3,330 | ≤10^†^ (0.3) | 3,328 | ≤10^†^ (0.3) | 1.33  (0.46-3.84) |
| Meningeal | 3,334 | 0 (0) | 3,334 | 0 (0) | n/a |
| Multiple myeloma | 3,328 | ≤10^†^ (0.3) | 3,327 | ≤10^†^ (0.3) | 0.17  (0.02-1.41) |
| **All-cause mortality** | 3,334 | 98 (2.9) | 3,334 | 206 (6.2) | 0.47  (0.37-0.60) |

**Abbreviations:** CI, confidence interval; CKD5, chronic kidney disease stage 5; ESRF, end stage renal failure; HCC, hepatocellular carcinoma; MALO, major adverse liver outcomes; MACE, major adverse cardiovascular outcomes; MAKE, major adverse kidney events; MALO, major adverse liver outcomes; MBS, metabolic bariatric surgery; MI, myocardial infarction; SLD, steatotic liver disease.

† To protect patients’ anonymity on TriNetX, if sample size is ≤10, the exact number of patients is concealed.

‡Intrahepatic cancer includes hepatocellular carcinoma and intrahepatic biliary tree cancers; intrahepatic cancer is a composite of MALO.


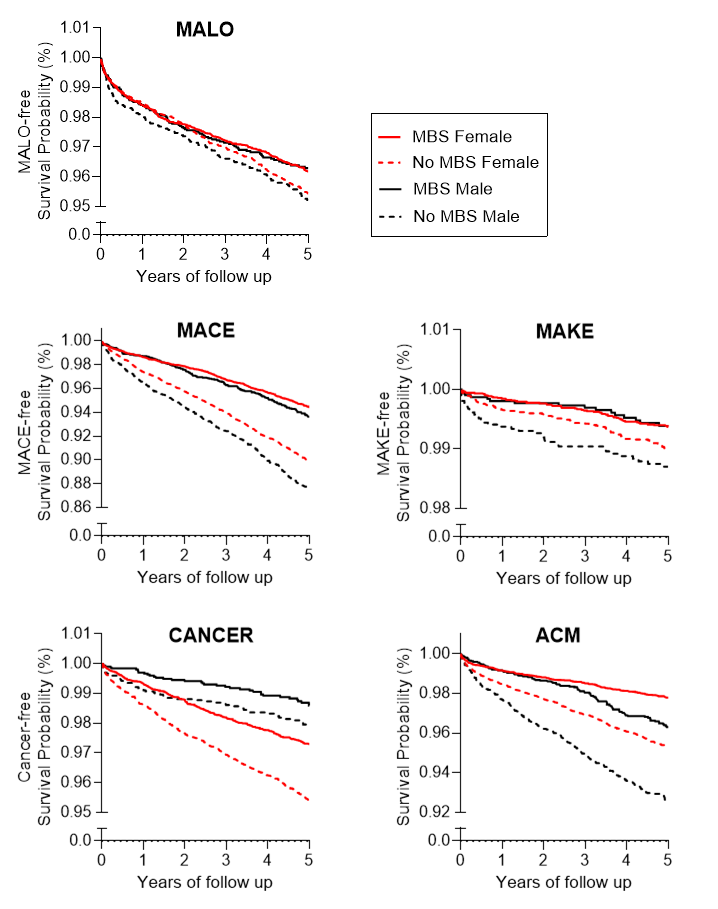


**Figure S5** Event-free probability for MBS and no-MBS cohorts in female and male patients. **Abbreviations**: ACM, all-cause mortality; CANCER, obesity-associated cancers; MACE, major adverse cardiovascular events; MAKE, major adverse kidney events; MALO, major adverse liver outcomes.

**Table S27** 30-day postoperative complications and mortality in the MBS cohort.

| **Complication** | **Number of patients with outcome** | **Absolute risk (%)** |
| --- | --- | --- |
| Any complication after MBS^†^ | 229 | 1.5 |
| Hemorrhage | 70 | 0.5 |
| Leak | 16 | 0.1 |
| Superficial Wound Infection | 10 | 0.1 |
| Venous Thromboembolism | 84 | 0.5 |
| Mortality | 25 | 0.2 |

^†^ Includes infectious and non-infectious complications with a generic code ‘complications after bariatric procedures’.
